# An adapted protocol to derive microglia from stem cells and its application in the study of CSF1R-related disorders

**DOI:** 10.1186/s13024-024-00723-x

**Published:** 2024-04-05

**Authors:** Marie-France Dorion, Diana Casas, Irina Shlaifer, Moein Yaqubi, Peter Fleming, Nathan Karpilovsky, Carol X.-Q. Chen, Michael Nicouleau, Valerio E. C. Piscopo, Emma J. MacDougall, Aeshah Alluli, Taylor M. Goldsmith, Alexandria Schneider, Samuel Dorion, Nathalia Aprahamian, Adam MacDonald, Rhalena A. Thomas, Roy W. R. Dudley, Jeffrey A. Hall, Edward A. Fon, Jack P. Antel, Jo Anne Stratton, Thomas M. Durcan, Roberta La Piana, Luke M. Healy

**Affiliations:** 1 Neuroimmunology Unit, Montreal Neurological Institute-Hospital, McGill University, Montreal, H3A 2B4 Canada; 2 Early Drug Discovery Unit, Montreal Neurological Institute-Hospital, McGill University, Montreal, H3A 2B4 Canada; 3 Department of Neurology and Neurosurgery, Montreal Neurological Institute-Hospital, McGill University, Montreal, H3A 2B4 Canada; 4 McGill Parkinson Program and Neurodegenerative Disorders Research Group, Montreal Neurological Institute-Hospital, McGill University, Montreal, H3A 2B4 Canada; 5 Faculty of Arts and Sciences, Université de Montréal, Montreal, H3T 1NB Canada; 6 Department of Pediatric Surgery, Division of Neurosurgery, Montreal Children’s Hospital, McGill University Health Centers, Montreal, H4A 3J1 Canada

**Keywords:** Microglia, iPSC, ALSP, CSF1R, Inflammation, Phagocytosis, Migration, CD68, P2RY12

## Abstract

**Background:**

Induced pluripotent stem cell-derived microglia (iMGL) represent an excellent tool in studying microglial function in health and disease. Yet, since differentiation and survival of iMGL are highly reliant on colony-stimulating factor 1 receptor (CSF1R) signaling, it is difficult to use iMGL to study microglial dysfunction associated with pathogenic defects in CSF1R.

**Methods:**

Serial modifications to an existing iMGL protocol were made, including but not limited to changes in growth factor combination to drive microglial differentiation, until successful derivation of microglia-like cells from an adult-onset leukoencephalopathy with axonal spheroids and pigmented glia (ALSP) patient carrying a c.2350G > A (p.V784M) *CSF1R* variant. Using healthy control lines, the quality of the new iMGL protocol was validated through cell yield assessment, measurement of microglia marker expression, transcriptomic comparison to primary microglia, and evaluation of inflammatory and phagocytic activities. Similarly, molecular and functional characterization of the ALSP patient-derived iMGL was carried out in comparison to healthy control iMGL.

**Results:**

The newly devised protocol allowed the generation of iMGL with enhanced transcriptomic similarity to cultured primary human microglia and with higher scavenging and inflammatory competence at ~ threefold greater yield compared to the original protocol. Using this protocol, decreased CSF1R autophosphorylation and cell surface expression was observed in iMGL derived from the ALSP patient compared to those derived from healthy controls. Additionally, ALSP patient-derived iMGL presented a migratory defect accompanying a temporal reduction in purinergic receptor P2Y12 (*P2RY12*) expression, a heightened capacity to internalize myelin, as well as heightened inflammatory response to Pam_3_CSK_4_. Poor P2RY12 expression was confirmed to be a consequence of *CSF1R* haploinsufficiency, as this feature was also observed following CSF1R knockdown or inhibition in mature control iMGL, and in *CSF1R*^*WT/KO*^ and *CSF1R*^*WT/E633K*^ iMGL compared to their respective isogenic controls.

**Conclusions:**

We optimized a pre-existing iMGL protocol, generating a powerful tool to study microglial involvement in human neurological diseases. Using the optimized protocol, we have generated for the first time iMGL from an ALSP patient carrying a pathogenic *CSF1R* variant, with preliminary characterization pointing toward functional alterations in migratory, phagocytic and inflammatory activities.

**Graphical Abstract:**

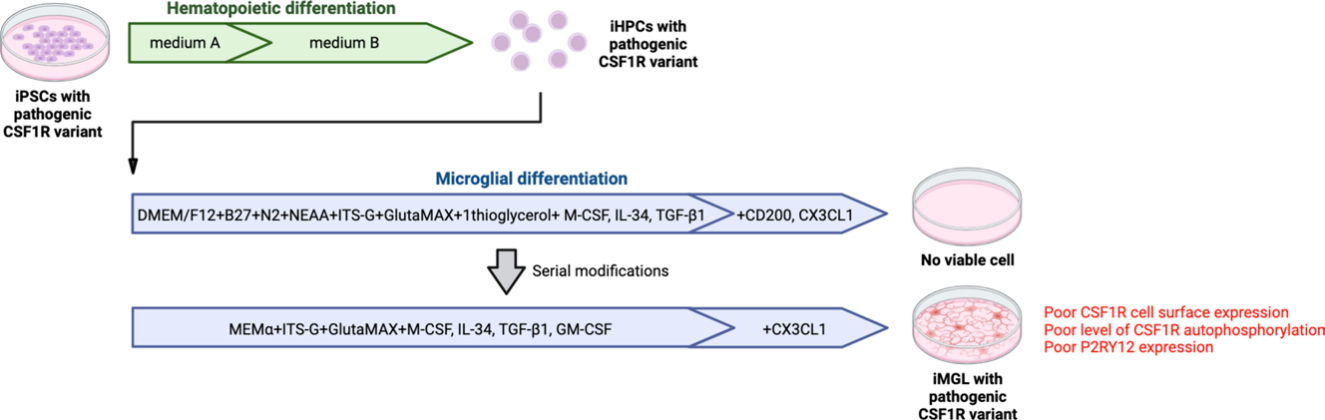

**Supplementary Information:**

The online version contains supplementary material available at 10.1186/s13024-024-00723-x.

## Background

Microglia are resident myeloid cells of the central nervous system that originate from yolk sac progenitor cells. Following brain colonization, microglia aid in the development of neuronal networks and myelination. As mononuclear phagocytes, they have crucial functions in the removal of excess neurons and synapses during development, as well as in the clearance of dying cells and debris. These highly motile cells express a diverse range of immune sensing receptors to act as sentinel cells of the brain parenchyma [1].

Recent advances in the human microglia field have been driven by the development of several induced pluripotent stem cell-derived microglia (iMGL) protocols starting in 2017 [2]. Microglial cells are generally derived from induced pluripotent stem cells (iPSCs) in two steps: 1) production of hematopoietic precursor cells, and 2) microglial differentiation. In particular, the iMGL protocol developed by Abud et al*.* [3] and later simplified by McQuade et al*.* (hereafter referred to as version “2.0” [4]) relies on the use of macrophage colony-stimulating factor (M-CSF), interleukin-34 (IL-34) and transforming growth factor-beta 1 (TGF-β1) for microglial differentiation. These growth factors are important for the acquisition of a unique molecular identity that distinguishes microglia from other myeloid populations [5–7].

M-CSF and IL-34 are endogenous ligands of the receptor tyrosine kinase colony-stimulating factor 1 receptor (CSF1R) essential for both the development and the maintenance of microglia pool in the brain through self-renewal [7–11]. In mice, *Csf1r* knockout results in an almost complete failure of microglia development [12]. In human, mono-allelic pathogenic variants in the *CSF1R* gene have been associated with adult-onset leukoencephalopathy with axonal spheroids and pigmented glia (ALSP). This rare autosomal dominant disease is pathologically characterized by vacuolating and demyelinated white matter especially in the frontal regions and corpus callosum, axonal degeneration and swelling (spheroids), and pigmented myeloid cells in the brain. Affected individuals present with psychiatric, cognitive and motor symptoms usually in the 4th decade, and rapidly deteriorate to death on average 7 years after disease onset [13, 14]. The impact of *CSF1R* pathogenic variants on microglia function and brain abnormalities remains poorly understood, and no treatment exists to date.

As most iMGL protocols including the 2.0 protocol developed by McQuade et al*.* heavily rely on CSF1R signaling for successful microglia differentiation [4] and survival [15], it did not appear possible to actively study *CSF1R*-mutated microglia using such protocols. We made serial modifications to the microglia differentiation medium used in the 2.0 protocol to ensure successful derivation of microglia-like cells from an ALSP patient harboring a pathogenic c.2350G > A *CSF1R* variant. This protocol, which conserved the simplicity of the 2.0 protocol all the while improving the overall functional competence of the resulting cells, will be referred to as the “2.9” protocol since it was the 9th iteration of the 2.0 protocol.

Herein, we present the new 2.9 protocol and the characterization of the resulting cells (“iMGL 2.9”) in comparison to cells generated using the original protocol (“iMGL 2.0”) and primary human microglia. This new protocol was used to carry out the cellular and molecular phenotyping of ALSP patient-derived iMGL carrying a heterozygous pathogenic variant in the *CSF1R* gene, which we failed to achieve using the 2.0 protocol due to unsuccessful differentiation.

## Methods

### iPSC lines

Characteristics of iPSC lines used in this study are presented in Additional file 3: Table S1. Generation of iPSC lines was done following McGill University Health Center’s ethical guidelines (project# 2019–5374) with written consent from donors. Some cell lines used in the analyses presented in this article were obtained from the Golub Capital iPSC Parkinson’s Progression Markers Initiative (PPMI) Sub-study (https://www.ppmi-info.org/access-data-specimens/request-cell-lines). As such, the investigators within PPMI contributed to the design and implementation of PPMI and/or provided data and collected samples but did not participate in the analysis or writing of this manuscript. For up-to-date information on the study, please visit PPMI-info.org. Cells were maintained in mTeSR™ Plus (STEMCELL Technologies) or in Essential 8™ media on Corning™ Matrigel™ hESC-Qualified Matrix -coated dishes, with subculturing every five to seven days using standard protocols [16].

For the generation of iPSCs from a *CSF1R*-mutated ALSP patient, peripheral blood mononuclear cells (PBMCs) were collected through the intermediary of C-BIG Repository, Montreal, Canada, with written consent from the donor and following McGill University Health Centre’s ethical guidelines. PBMCs were reprogrammed into iPSCs using previously established episomal reprogramming method [16]. Briefly, PBMCs were nucleofected with episomal plasmids (pEV-OCT4-2A-SOX2, pEV-MYC, pEV-KLF4, and pEV-BCL-XL) and resulting iPSC colonies were selected based on the acquisition of stem cell-like morphology for expansion and cryopreservation. Genomic integrity was then verified by karyotyping and quantitative polymerase chain reaction (qPCR)-based assays as previously described [16].

### Generation of iMGL

*Hematopoietic differentiation.* Differentiation of iPSCs into iPSC-derived hematopoietic progenitor cells (iHPCs) was carried out using STEMdiff™ Hematopoietic kit (STEMCELL Technologies) as previously described [4] with minor modifications. On day -1, iPSCs were detached using Gentle Cell Dissociation Reagent (STEMCELL Technologies) and plated in Matrigel™-coated 6-well plates, in mTeSR™ Plus or in Essential 8™ media supplemented with Y27632 (10 μM, Selleckchem). Several seeding densities should be tested for every differentiation batch, aiming in the range of 1–5 small colonies per cm^2^ at the start of the differentiation. On day 0, media were replaced with STEMdiff™ hematopoietic medium A (2 mL/well). On day 2, half the volume of the cell supernatants (1 mL/well) was replaced with fresh medium A. On day 3, media was fully replaced with STEMdiff™ hematopoietic medium B (2 mL/well). On day 5 and 7, half the volume of the cell supernatants (1 mL/well) was replaced with fresh medium B. On day 9, 1 mL/well of fresh medium B was added. On day 10, cell supernatants containing the floating iHPCs were collected and spun down at 300 g for 5 min. 1 mL/well of cell-free conditioned media, along with 1 mL/well of fresh medium B, were put back on the iHPC culture. The collected pellet of iHPCs was processed either for cryopreservation (using Bambanker, Fujifilm Wako Chemicals) or for microglial differentiation. iHPCs were similarly harvested on day 12. Each harvest should result in the collection of 0.1—1 × 10^6^ iHPCs/well. Although iHPCs could also be collected on day 14, iHPCs from day 10 and day 12 showed higher cell proliferation during microglial differentiation (personal observation).

*Microglial differentiation.* On day 0, iHPCs were resuspended at a density of 5–10 × 10^5^ cells/mL in microglia differentiation medium 2.0 or 2.9 (Table 1) and plated on Matrigel™-coated 6-well plates (2 mL/well). The culture was supplemented with 1 mL/well of differentiation media every other day. Every 10 to 12 days, 5 mL/well of cell supernatants were collected and spun down at 300 g for 5 min. The collected cells were resuspended in 1 mL/well of differentiation media and placed back in culture. Cells were considered mature on day 28 of microglial differentiation, with downstream experiments carried out between day 28 and 42, unless otherwise specified. Cells were maintained at 37 °C under a 5% CO2 atmosphere. For any downstream experiments, cells were detached using phosphate-buffered saline (PBS) with 2 mM ethylenediaminetetraacetic acid (EDTA; 10-min incubation) and replated at a density of 7.5 × 10^5^ cells per cm^2^.

**Table 1:** Composition of the 2.0 and 2.9 microglia differentiation media

2.0 microglia differentiation medium	2.9 microglia differentiation medium	Catalog number
DMEM/F12	-	11039047 (Thermo Fisher Scientific)
-	MEMα	12571063 (Thermo Fisher Scientific)
GlutaMAX 1X	GlutaMAX 1X	35050061 (Thermo Fisher Scientific)
Non-essential amino acids 1X	-	11140050 (Thermo Fisher Scientific)
B27 2X	B27 2X	17504044 (Thermo Fisher Scientific)
N2 0.5X	-	17502048 (Thermo Fisher Scientific)
Insulin-Transferrin-Selenium 2X	Insulin-Transferrin-Selenium 2X	41400045 (Thermo Fisher Scientific)
Monothioglycerol 400 μM	-	88640 (Sigma-Aldrich)
Penicillin/Streptomycin 1X	Penicillin/Streptomycin 1X	15140122 (Thermo Fisher Scientific)
IL-34* 100 ng/mL	IL-34* 100 ng/mL	200–34 (Peprotech)
TGF-β1* 50 ng/mL	TGF-β1* 50 ng/mL	100–21 (Peprotech)
M-CSF* 25 ng/mL	M-CSF* 25 ng/mL	300–25 (Peprotech)
-	GM-CSF* 5 ng/mL	300–03 (Peprotech)
CX3CL1*^,#^ 100 ng/mL	CX3CL1*^,#^ 100 ng/mL	300–18 (Peprotech)
CD200*^,#^ 100 ng/mL		174024 (Abcam)

^*^Freshly added, #from day 24 onward

### Primary microglia isolation and culture

Human brain tissues from 2- to 65-year-old female and male epilepsy patients were obtained from the Montreal Neurological Institute, Montreal, Canada (adult donors) and the Montreal Children's Hospital, Montreal, Canada (pediatric donors), with written consent and under local ethic boards’ approval. Tissues were from sites distant to the suspected primary epileptic foci. Isolation of glial cells was carried out as previously described [17] through mechanical and chemical digestion, followed by Percoll® (Sigma-Aldrich) gradient centrifugation. Microglia were further purified by taking advantage of the differential adhesive properties of the glial cells. Cells were maintained in Minimum Essential Medium (MEM; Sigma-Aldrich) supplemented with 5% fetal bovine serum (FBS; Wisent Bio Products), 1% P/S, 0.1% glucose and 1X GlutaMAX™, unless otherwise specified. Fetal microglia were isolated similarly [17] from second trimester fetal brain tissues obtained from Centre Hospitalier Universitaire Sainte-Justine, Montreal, Canada, with maternal written consent and under local ethic boards’ approval. Fetal microglia were cultured in Dulbecco’s Modified Eagle’s Medium (DMEM; Thermo Fisher Scientific) supplemented with 5% FBS, 1% P/S and 1X GlutaMAX™.

### Peripheral blood mononuclear cell-derived macrophages (PBMC-Mφ)

PBMCs were isolated from whole blood by Ficoll gradient centrifugation. Monocytes were then collected through magnetic activated bead sorting of cluster of differentiation 11b (CD11b) -positive cells, and cultured at a density of 5–10 × 10^5^ cells/cm^2^ (day 0) in RPMI-1640 (Thermo Fisher Scientific) with 10% FBS, 1% P/S, 1X GlutaMAX™ and 30 ng/mL M-CSF (Peprotech). Cells were matured for 8 days, with media supplementation on day 4 and day 7.

### Immortalized cell lines

hTERT RPE-1 and THP-1 cells were from American Type Cell Collection. hTERT RPE-1 cells were maintained in DMEM/F12 with 10% FBS and 1% P/S with subculturing every four days. THP-1 cells were maintained in RPMI-1640 with 10% FBS, 1% P/S and 1X GlutaMAX™ with subculturing every five days.

### Adhesion assay

A crystal violet kit (Abcam) was used to assess cell adhesion following the manufacturer’s protocol. Cells were first seeded on a 96-well plate. After 48 h, cells were washed with washing buffer to remove non-adherent cells, prior to incubation with crystal violet. After washing off the excess crystal violet, cells were incubated in the solubilization buffer. Absorbance was measured at 560 nm on a SpectraMax® iD3 microplate reader (Molecular Devices).

### Immunocytochemistry

Cells were fixed in 4% paraformaldehyde solution and permeabilized/blocked using PBS with 3% goat or donkey serum and 0.2% triton X-100 (Sigma Aldrich). Cells were incubated at 4 °C overnight with primary antibodies against the following targets: ionized calcium binding adaptor molecule 1 (IBA1; #NC9288364, Fujifilm Wako Chemicals at 1:1000), PU.1 (#2258, Cell Signaling at 1:250), Nanog (#ab21624, Abcam at 1:500), Tra-1–60 (#60064, STEMCell Technologies at 1:200), stage-specific embryonic antigen-4 (SSEA-4; #sc-21704, Santa Cruz Biotechnologies at 1:200), octamer-binding transcription factor 3/4 (OCT3/4; #sc-8628, Santa Cruz Biotechnologies at 1:500), Ki67 (#556003, BD Biosciences at 1:200), lysosomal-associated membrane protein 1 (LAMP1; #9091, Cell Signaling at 1:200) or cluster of differentiation 68 (CD68; #M0814, Dako Omnis at 1:200). Cells were then incubated with secondary antibodies and 1 μg/mL Hoechst 33,342 for one hour. The proportion of cells with positive stain was determined using a CellInsight CX5 High Content Screening Platform (Thermo Fisher Scientific). All conditions were assessed in triplicate.

### Ribonucleic acid (RNA) -sequencing

TRIzol (Thermo Fisher Scientific) was used to extract RNA, followed by cleaning using a RNeasy mini kit (Qiagen). Quality control of the RNA samples, as well as the library preparation by poly(A) enrichment and RNA-sequencing were performed by Genome Quebec, Montreal, Canada. RNA-sequencing was performed using an Illumina NovaSeq 6000 with a read depth of 50 million reads per sample. Canadian Center for Computational Genomic’s pipeline GenPipes [18] was used to align the raw files and quantify the read counts. Briefly, raw fastq files were aligned to the GRCh38 genome reference using STAR aligner [19] with default parameters and raw reads were quantified using HTseq count [20]. Differential expression gene (DEG) analysis was carried out using the DESeq2 package [21]. DEGs were identified using an adjusted p-value cutoff of 0.05. Gene ontology (GO) enrichment analyses were performed using PANTHER overrepresentation test, on the web-based tool offered by the Gene Ontology Consortium. Principal component analysis (PCA) was carried out using the Python module sklearn and visualized using Matplotlib. Histograms were generated using GraphPad Prism 9.0 software.

RNA-sequencing data from Douvaras et al*.,* 2017 [22], Konttinen et al*.,* 2019 [23], and Drager et al*.,* 2022 [24] were obtained from Gene Expression Omnibus (GSE97744, GSE135707, and GSE178317, respectively). Batch effect correction was performed using the sva package [25].

### Quantitative reverse transcription polymerase chain reaction (qRT-PCR)

Following RNA extraction, reverse transcription was performed using Moloney murine leukemia virus reverse transcriptase (Thermo Fisher Scientific). Real-time PCR was performed using TaqMan assays (Thermo Fisher Scientific) on a QuantStudio™ 5 real-time PCR system (Thermo Fisher Scientific). The 2^−ΔCt^ method was used to analyze the data using glyceraldehyde 3-phosphate dehydrogenase (*GAPDH*) and tyrosine 3-monooxygenase/tryptophan 5-monooxygenase activation protein zeta (*YWHAZ*) as controls.

### Flow cytometry

Cells were blocked with Human TrueStain FcX and TrueStain Monocyte Blocker (Biolegend) and stained with the following antibodies: anti-cluster of differentiation 34 (CD34; clone #561, Biolegend), anti-cluster of differentiation 43 (CD43; clone #CD43-10G7, Biolegend), anti-cluster of differentiation 14 (CD14; clone #HCD14, Biolegend), anti-CSF1R (clone #61708, R&D Systems), anti-CX-3-C motif chemokine receptor 1 (CX3CR1; clone #2A9-1, Biolegend), anti-Mer tyrosine kinase (MERTK; clone #125518, R&D Systems), anti-purinergic receptor P2Y12 (P2RY12; clone #S16001E, Biolegend) or anti-toll-like receptor 4 (TLR4; clone #610029, R&D Systems). All antibodies were titrated using negative control cells that don’t or poorly express the target protein. For intracellular staining, cells were fixed in 4% paraformaldehyde solution and permeabilized in 0.2% triton X-100 solution prior to antibody staining. Appropriate forward and side scatter profiles were used to exclude debris and doublets from the analysis. Dead cells were excluded based on LIVE/DEAD™ Fixable Aqua (Thermo Fisher Scientific) staining. Readings were done on an Attune™ Nxt Flow Cytometer and analyzed/visualized using FlowJo™ software.

### Phagocytosis assay

Human α-synuclein preformed fibrils [26], myelin debris [27] and immunoglobulin G-opsonized red blood cells [28] were labelled with pHrodo™ Green STP ester or pHrodo™ Red succinimidyl ester (Thermo Fisher Scientific) as previously described and used at the following respective concentrations which were determined to be non-saturating: 1 μM, 15 μg/mL and 5 × 10^4^ cells/mL. Bioparticles of pHrodo™ Green-labelled *Escherichia coli* (*E. coli*) were purchased from Thermo Fisher Scientific and used at a concentration of 25 μg/mL. Cells were incubated with the labelled substrates for three hours unless otherwise indicated, and before being counterstained with Hoechst 33342 (5 μg/mL). Total green fluorescence intensity per cell was quantified on a CellInsight CX5 High Content Screening Platform. All conditions were assessed in triplicate. Unchallenged cells were used to measure background/autofluorescence. Histograms were generated using the Python library Matplotlib. Internalization of fluorescein isothiocyanate (FITC) -labelled myelin was assessed similarly, except cells were washed with 0.4% trypan blue solution to quench extracellular fluorescence prior to imaging.

### Measurement of cytokine secretion

The following reagents were used to treat cells: lipopolysaccharide (LPS) from *E. coli* strain O127:B8 (100 ng/mL; Sigma Aldrich), Pam_3_CSK_4_ (100 ng/mL; Invivogen), R-FSL-1 (250 ng/mL; EMC Microcollection Gmbh), interferon gamma (IFNγ; 10 ng/mL; Peprotech) and adenosine triphosphate (ATP; 5 mM; Sigma Aldrich). Concentrations of interleukin-1beta (IL-1β), interleukin-6 (IL-6), interleukin-10 (IL-10) and tumor necrosis factor (TNF) in cell supernatants were measured using the Human Inflammatory Cytokine Cytometric Bead Array Kit (BD Biosciences). Concentrations of chemokines in cell supernatants were measured using the LEGENDplex™ Human Proinflammatory Chemokine Panel 1 (Biolegend). Readings were made on an Attune™ Nxt Flow Cytometer.

### Western blotting

Cells were lysed on ice in a lysis buffer composed of 150 mM NaCl, 50 mM Tris–HCl pH 7.4, 1% Nonidet P-40, 0.1% sodium dodecyl sulfate (SDS) and 5 mM EDTA with protease and phosphatase inhibitors (Thermo Fisher Scientific). Cell lysates were centrifuged at 500 g for 30 min at 4 °C to remove cellular debris. Proteins (25 μg/lane) were separated on SDS–polyacrylamide gels and transferred to polyvinylidene difluoride membranes (Bio-Rad Laboratories). Membranes were immunoblotted for CSF1R (1:250; #MAB3291, R&D Systems) and its phosphorylated form (Y723; 1:500; #3155, Cell Signaling), nuclear factor kappa B (NF-κB; 1:500; #8282, Cell Signaling) and its phosphorylated form (S536; 1:500; #3033, Cell Signaling), caspase-1 (1:500; #ab179515, Abcam), IL-1β (1:500; #12,242, Cell Signaling), NLR family pyrin domain containing 3 (NLRP3; 1:500; #15101S, Cell Signaling) and GAPDH (1:5000; G8795, Sigma Aldrich) overnight at 4 °C, and then with horse radish peroxidase-linked secondary antibodies (1:10,000; Jackson Laboratory) for one hour. Bands were detected by enhanced chemiluminescence SignalFire™ Plus ECL reagent (Cell Signaling) using a ChemiDoc Imaging System (Bio-Rad Laboratories). Image analysis was performed using ImageLab 6.0.1 software (Bio-Rad Laboratories).

### *CSF1R* sequencing

*CSF1R* pathogenic variant (c.2350G > A; p.V784M) in ALSP patient’s PBMCs and iPSCs was confirmed by Sanger sequencing. Following DNA extraction, touchdown polymerase chain reaction (PCR) was performed using the forward and reverse primers 5’ACGATACACATTCTCAGATCCTGG 3’ and 5’GTGTAGACACAGTCAAAGATGCTC 3’ respectively, for PBMCs, and 5’GGTAGGAGAAGGCCCAAGAC 3’ and 5’GGGATGACAGTCCCCAGTTA 3’, respectively, for iPSCs (designed using NCBI’s primer design tool, https://www.ncbi.nlm.nih.gov/tools/primer-blast/, and NM_005211.4 as reference sequence). The optimal annealing temperature for the primers was established at 54 °C using the Tm Calculator provided by New England BioLabs (https://tmcalculator.neb.com/#!/main). The amplified deoxyribonucleic acid (DNA) sample corresponding to the PBMCs was sent to Genome Quebec, Montreal, Canada, for Sanger sequencing performed on an Applied Biosystems 3730xl DNA Analyzer (Thermo Fisher Scientific). For the iPSC-derived sample, sequencing was performed on an Applied Biosystems SeqStudio Genetic Analyzer (Thermo Fisher Scientific).

Sequencing of the entire region encoding the tyrosine kinase domain of CSF1R was performed for iMGL using ENST00000675795.1 as the reference transcript sequence. Following RNA extraction and reverse transcription, complementary DNA (cDNA) was subjected to amplification cycles using three sets of primers (Additional file 1: Figure S1A, B). The optimal annealing temperature for the primers was established at 52 °C using the Tm Calculator provided by New England BioLabs (https://tmcalculator.neb.com/#!/main). PCR products were purified using an ExoSAP-IT PCR product cleanup reagent (Thermo Fisher Scientific). DNA sequencing reactions were performed using a BigDye v3.1 cycle sequencing kit (Thermo Fisher Scientific) and followed by purification using a BigDye XTerminator Purification kit (Thermo Fisher Scientific). Sequencing was carried out on an Applied Biosystems SeqStudio Genetic Analyzer (Thermo Fisher Scientific).

### Magnetic resonance imaging (MRI)

Brain MRI was clinically performed in the 1.5 T Philips MR scanner of the Montreal Neurological Institute, Montreal, Canada, with a protocol including 3D FLAIR T2-weighted images, axial T2-weighted images and diffusion weighted images.

### Propidium iodide (PI) staining

Cells were stained in PBS containing 1 μg/mL PI (Thermo Fisher Scientific) and 5 μg/mL Hoechst 33342. The average number of PI- live cells per condition was determined using a CellInsight CX7 High Content Screening Platform. All conditions were assessed in triplicate.

### Migration assay

A Boyden chamber assay was carried out to assess cell migration toward adenosine diphosphate (ADP). Cells were plated on top compartments of Corning® Transwell® inserts with 8.0 μm pores (#3422), in nucleoside-free MEMα. Bottom compartments were filled with nucleoside-free MEMα containing vehicle or ADP (20 μM). When indicated, PSB0739 (20 μM) was added to both top and bottom compartments. Migration was quantified 1.5 h later through Hoechst 33342 staining (5 μg/mL) of cells that crossed the inserts toward the lower compartment, using an EVOS M5000 Imaging System (Thermo Fisher Scientific).

### Assessment of lysosomal pH

Cells were incubated for one minute with 2.5 μM LysoSensor™ Yellow/Blue DND-160 (Thermo Fisher Scientific). Fluorescence intensity was assessed using a SpectraMax® iD3 microplate reader, at excitation wavelengths of 329 and 384 nm, and emission wavelength of 540 nm. Unstained cells were used for background subtraction. Fluorescence intensity ratio (Ex 329:384) was calculated as an indicator of lysosomal pH as previously described [29]. All conditions were assessed in triplicate. When indicated, cells were treated with 50 mM ammonium chloride (Sigma Aldrich) for one hour prior to the staining, throughout the staining, and during fluorescence intensity measurement.

### Assessment of lysosomal protease activity

Cells were incubated with DQ™ red bovine serum albumin (BSA) following the manufacturer’s recommendation for 24 h. Total red fluorescence intensity per cell was quantified using a CellInsight CX5 High Content Screening Platform. All conditions were assessed in triplicate. Unchallenged cells were used to measure background/autofluorescence. When indicated, cells were concomitantly treated with 100 nM Bafilomycin A1 (Thermo Fisher Scientific).

### Clustered regularly interspaced palindromic repeats (CRISPR) -Cas9-mediated gene editing

CRISPR-Cas9 gene editing approach was employed as previously described [30] to edit the *CSF1R* gene in the healthy control iPSC line 3450 (Additional file 3: Table S1). Briefly, iPSCs were nucleofected with ribonucleoprotein complex which contained Cas9 protein, single guide RNA (sequence: UGUUACGCGCUGCCACGUCC) and homology DNA repair template (sequence: CCTGCAGTGCTTTCCCTCAGTGCATCCACCGGGACGTGGCAGCGCGTAACATGCTGTTGACCAATGGTCATGTGGCCAAGATTGGGGACTTCGGGCTGGCT) using a Lonza 4D-Nucleofector device. Following limiting dilution, clones with edited *CSF1R* were identified by droplet digital PCR (QX200™ Droplet Reader, Bio-Rad) and Sanger sequencing.

### RNA interference

Cells were transfected with 10 nM siGENOME RISC-Free Control (siCON) or ON-TARGETplus small interfering RNA (siRNA) pools against *CSF1R* (siCSF1R; Horizon Discovery) using Lipofectamine™ RNAiMAX (Thermo Fisher Scientific). The manufacturers’ protocols were followed.

### Plasmids and lentivirus production

8 × 10^6^ HEK293T cells (American Cell Type Collection) were seeded into a 150-mm dish in DMEM supplemented with 10% FBS. After 24 h, the cells were transfected using X-tremeGENE™ 9 DNA Transfection Reagent (Roche) with the following plasmids in equal molar ratio (total 16 µg DNA): psPAX2 (#12260, Addgene), pMD2.G (#12259, Addgene), and WT CSF1R-IRES2-eGFP (EX-A3543-Lv165, Genecopoeia) or 2350G > A CSF1R-IRES2-eGFP (CS-A3543-Lv165-01, Genecopoeia). NM_005211.4 was used as the reference sequence. Media was changed to Opti-MEM (Thermo Fisher Scientific) 16 h post-transfection. Media was collected 48 h post-transfection, filtered (0.45 μM), and applied to iHPCs (500 uL/0.25 × 10^6^ cells) in the 2.9 medium. Media of iHPCs was fully replaced the next day and the cells were differentiated for 28–42 days. Transduction efficiency was assessed by eGFP fluorescence using an Attune™ Nxt Flow Cytometer.

### Statistical analyses

Statistical analyses were performed using GraphPad Prism 9.0 software. A t-test was used to compare the mean of two groups of data. A one-way analysis of variance (ANOVA) was used to compare the mean of three or more groups of data. When the assumptions of a t-test or a one-way ANOVA were not met, a Mann–Whitney test or a Kruskal–Wallis test was used instead, respectively. A two-way ANOVA was used to compare two groups of data with multiple variables. *P*-values (‘p’) were adjusted using appropriate post hoc tests following one-way and two-way ANOVA, and Kruskal–Wallis tests. Mean and standard error of the mean (SEM) of biological replicates (‘n’) are plotted in all graphs unless otherwise indicated. A *p* < 0.05 was considered statistically significant. Primary microglia obtained from the same donor or iMGL from the same differentiation batch divided into several cell culture wells were considered technical replicates. Primary microglia obtained from independent donors and iMGL generated at different points in time were considered biological replicates.

## Results

### The 2.9 protocol yields higher number of pure, adherent iMGL compared to the 2.0 protocol

**Fig. 1:**
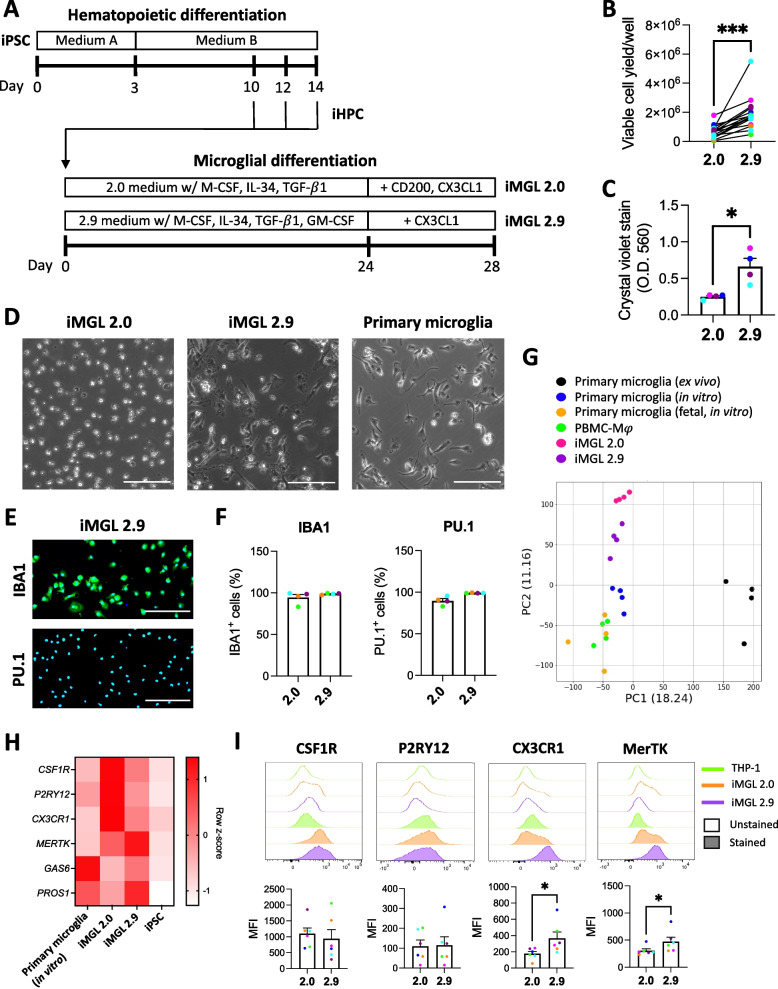
Characterization of iMGL 2.9. **A** Schematic of the 2.0 and 2.9 protocols. **B**-**H** iMGL 2.0 and 2.9 differentiation were carried out side-by-side from the same healthy control iPSC lines. **B** Viable iMGL cell yield assessed by trypan blue exclusion assay. Cell numbers per well of a 6-well plate are presented. A t-test was performed. *n* = 13 differentiation batches from 6 iPSC lines. *** *p* < 0.001. Connecting lines show side-by-side experiments. **C** Crystal violet assay. A Mann–Whitney test was performed. *n* = 4 lines, * *p* < 0.05, O.D. = optical density. **D** Phase contrast images of iMGL 2.0, iMGL 2.9 and primary human microglia. Scale bar = 150 $\mu m$. **E** Representative images of IBA1 and PU.1 immunostaining. Blue = Hoechst 33342, green = IBA1 or PU.1, scale bar = 200 μm. **F** Quantification of IBA1- and PU.1-immunopositivity. *n* = 4 lines. **G** PCA plot of RNA-sequencing data. **H** Heatmap showing key microglia marker expression assessed by qRT-PCR. *n* = 4 donors for primary microglia, 6 lines for iMGL 2.0/2.9 and iPSCs. **I** Flow cytometry assessment of cell surface microglia marker expression in iMGL 2.0 and 2.9. Data from THP-1 cells are shown as negative controls. Mann–Whitney tests were performed. *n* = 6 lines, * *p* < 0.05. MFI = median fluorescence intensity

The newly devised 2.9 protocol consisted of two steps (Fig. 1A): 1) generation of iHPCs from iPSCs identically to McQuade et al.’s 2.0 protocol using a commercially available kit, and 2) differentiation of iHPCs into iMGL using a modified medium formulation from the 2.0 protocol (Table 1). High glucose concentrations can mask cellular phenotype resulting from genetic manipulations or variants, especially if it relates to cellular metabolism [31]. Furthermore, energy metabolism is intricately linked to immune function of microglia [32, 33], with glucose concentration influencing microglial inflammatory response to LPS [34]. MEMα containing physiological concentration of glucose (5.6 mM) was therefore used as the base of the 2.9 microglia differentiation medium, instead of DMEM/F12 in the 2.0 medium which contains three times higher concentration of glucose (17.5 mM). Since MEMα contains optimal concentrations of non-essential amino acids (glycine, alanine, asparagine, aspartic acid, glutamic acid, proline, and serine) contrary to DMEM/F12, the 2.9 medium was not further supplemented with non-essential amino acids. The growth factor granulocyte–macrophage colony-stimulating factor (GM-CSF) at low concentration has been previously shown to be beneficial in increasing iMGL cell yield [35] and was therefore incorporated as a mitogenic factor, in addition to IL-34, M-CSF and TGF-β1. While C-X3-C motif chemokine ligand 1 (CX3CL1) and cluster of differentiation 200 (CD200) had been used in the 2.0 protocol as modulators of microglial function, CD200 was omitted in the 2.9 protocol as the expression of its receptor is almost absent in human microglia [15, 36]. Finally, monothioglycerol and N2 supplements were omitted, as they have been shown to provide no benefit in microglia identity acquisition [35].

From the same starting number of iHPCs (0.1—0.2 × 10^6^/well), the new 2.9 protocol resulted on average in ~ 3 times higher number of viable iMGL (~ 2 × 10^6^/well) compared to the 2.0 protocol (~ 0.7 × 10^6^/well; Fig. 1B), with some variability in yield improvement observed between different iPSC lines (between 2 to eightfold increase; Additional file 1: Figure S2A). Line-to-line variability in cell yield has also been previously observed with various iMGL protocols [37–39]. Cell source, reprogramming method and sex of iPSCs did not appear to influence final iMGL 2.9 cell yield (Additional file 1: Figure S2B). In addition to successful differentiation of control iPSC lines into iMGL, the 2.9 protocol was also confirmed to allow the differentiation of Parkinson’s disease patient-derived lines harboring glucocerebrosidase (*GBA*) or leucine-rich repeat kinase 2 (*LRRK2*) pathogenic variants (Additional file 1: Figure S3A-B). In contrast to the 2.0 protocol that yielded loosely adherent and floating cells, the 2.9 protocol resulted in more tightly adherent cells of elongated, amoeboid or ramified morphologies akin to primary cells (Fig. 1C-D, Additional file 1: Figure S4). Progressive acquisition of these microglial morphologies and adhesion to cultureware were observed throughout the differentiation, as soon as on day 2 (Additional file 1: Figure S5A). Occasional formation of multinucleated giant cells could be observed (Additional file 1: Figure S6A), but those could be eliminated through the selective harvest of mononuclear cells using a 2 mM EDTA solution (Additional file 1: Figure S6B). When replated for downstream experiments, iMGL adhered to cultureware within 30 min, and regained microglia-like morphologies after 16 h (Additional file 1: Figure S5B). Immunostaining revealed mature iMGL 2.9 to be ~ 99% and ~ 98% positive for the respective myeloid markers IBA1 and PU.1 (Fig. 1E-F, Additional File 1: Figure S7A-B; the neuroepithelial cell line hTERT RPE-1 was used as a negative control).

RNA-sequencing revealed the transcriptomes of iMGL 2.0 and 2.9 to greatly differ from that of iPSCs from which they were generated, and to closely resemble that of cultured primary microglia (Additional file 1: Figure S8A), as well as the transcriptomes of iMGL preparations from earlier publications (Douvaras et al., 2017 [22], Konttinen et al., 2019 [23] and Drager et al., 2022 [24]; Additional file 1: Figure S8B). Transcriptomes of iMGL generated using either the 2.0 or 2.9 protocols slightly differed from cultured (in vitro) and freshly isolated (ex vivo) primary microglia, but iMGL 2.9 showed a higher transcriptomic similarity to in vitro and ex vivo primary microglia compared to iMGL 2.0 (Fig. 1G). PCA revealed a number of genes including *AXL*, allograft inflammatory factor 1 (*AIF1*, encoding IBA1) and toll-like receptor 10 (*TLR10*) to be aberrantly high in iMGL 2.0 compared to iMGL 2.9 and in vitro and ex vivo primary microglia (Additional file 1: Figure S9, Additional file 4: Table S2). DEG analysis revealed genes more highly expressed in iMGL 2.0 over 2.9 to be enriched in processes related to cell division (Additional file 1: Figure S10A). Genes more highly expressed in iMGL 2.9 over 2.0 were involved in microglial cell activation (*e.g.* triggering receptor expressed on myeloid cell 2 or *TREM2*, chemokine ligand 3 or *CCL3*, and metalloproteinase 8 or *MMP8*), chemotactic attraction of leukocytes and complement system (Additional file 1: Figure S10B). A number of macrophage-specific markers such as myeloperoxidase (*MPO*) were confirmed to be lowly expressed in both iMGL 2.0 and 2.9 compared to PBMC-Mφ (Additional file 1: Figure S11). Cluster of differentiation 36 (*CD36*) was previously described as a marker of fetal microglia [40] and was more highly expressed on cultured fetal microglia and PBMC-Mφ than postnatal microglia or iMGL 2.0 and 2.9 (Additional file 1: Figure S11). Genes that are known to be more highly expressed in microglia over macrophages such as *P2RY12*, G-protein coupled receptor 34 (*GPR34*) or sialic acid binding Ig-like lectin 10 (*SIGLEC10*) were more highly expressed in iMGL 2.9 compared to PBMC-Mφ (Additional file 1: Figure S11). Assessment of homeostatic microglia markers revealed some markers such as *CSF1R*, *P2RY12* and *CX3CR1* to be lower in iMGL 2.9 compared to iMGL 2.0, whereas other markers such as *MERTK*, growth arrest-specific 6 (*GAS6*) and protein S (*PROS1*) were higher in iMGL 2.9 (Fig. 1H, Additional file 1: Figure S11). However, flow cytometry assessment of cell surface expression revealed CX3CR1 and MerTK to be higher in iMGL 2.9 compared to iMGL 2.0, and CSF1R and P2RY12 to be similar between the two protocols (Fig. 1I; the monocytic cell line THP-1 was used as a negative control). Flow cytometry experiment in permeabilized cells revealed total CSF1R, P2RY12 and CX3CR1 protein expression to be higher in iMGL 2.9 compared to iMGL 2.0 (Additional file 1: Figure S12), suggesting higher protein expression is responsible for the discrepancy observed between transcriptional and cell surface protein expression data.

iMGL constitute a useful model in investigating the role of microglia in diseases. In particular, impact of disease-associated risk variants on microglial function can be studied using patient-derived or genetically modified lines. The expression of risk genes associated with Alzheimer’s disease [41] and Parkinson’s disease [42], first and second most common neurodegenerative disorders, in iMGL 2.9 was analyzed. RNA-sequencing showed clear similarity in the expression profile of risk genes between iMGL 2.0/2.9 and ex vivo primary microglia, which was distinctively different from the expression profile in iPSCs (Additional file 1: Figure S13). Some disease-associated risk genes such as apolipoprotein E (*APOE*), complement receptor 1 (*CR1*) or cathepsin B (*CTSB*) were more highly expressed in iMGL 2.9 and ex vivo primary microglia than in iMGL 2.0 (Additional file 1: Figure S13), suggesting iMGL 2.9 would be a better model than iMGL 2.0 in investigating the impact of variants affecting those genes.

Washer et al*.* recently published a protocol of iMGL generation in which Advanced DMEM/F12 with GlutaMAX™, M-CSF, IL-34, TGF-β1 and GM-CSF was used as the microglia differentiation medium. The equivalence of Washer et al*.*’s medium to the 2.9 medium was assessed (Additional file 1: Figure S14A). After 28 days of iHPC differentiation into iMGL using Washer et al*.*’s medium, formation of cell clusters (Additional file 1: Figure S14B) with poor expression of microglia markers was observed, compared to cells differentiated using the 2.0 or the 2.9 medium (Additional file 1: Figure S14C). This suggests microglial differentiation medium cannot be interchangeably used to differentiate distinct precursor cell preparations into microglial cells.

Overall, our findings imply that relative to the 2.0 protocol, the 2.9 protocol results in an improved yield of more adherent microglia-like cells with higher transcriptomic similarity to cultured primary microglia.

### iMGL 2.9 are better phagocytes than iMGL 2.0

**Fig. 2:**
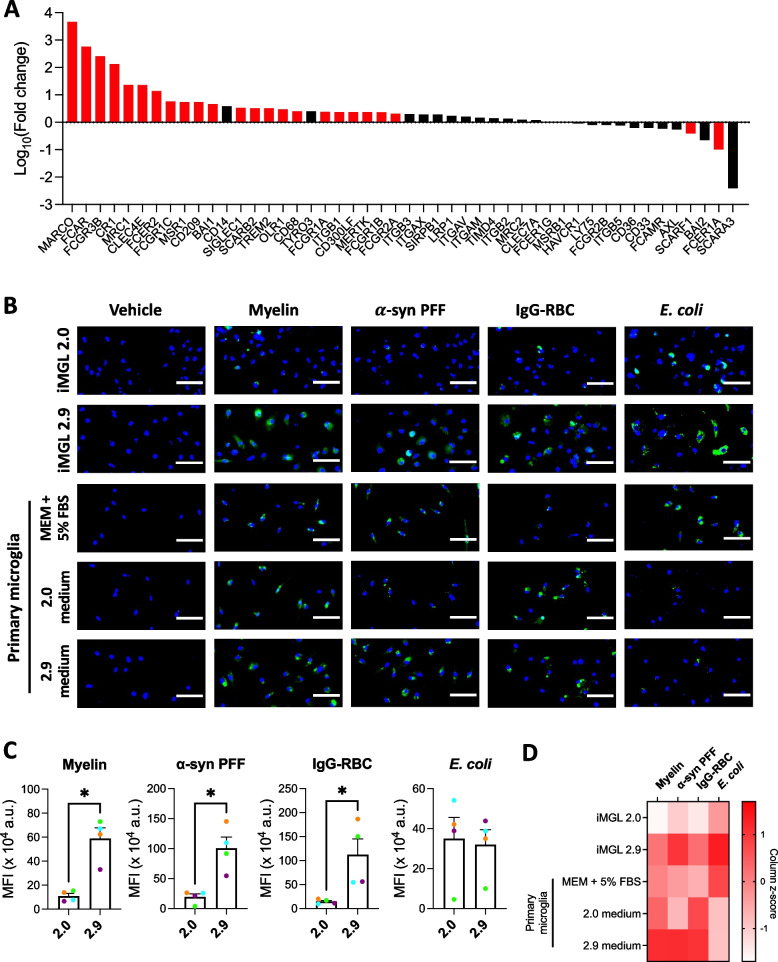
Scavenging abilities of iMGL 2.9. iMGL 2.0 and 2.9 were differentiated side-by-side from the same healthy control iPSC lines. **A** Expression of genes encoding phagocytosis/scavenger receptors or positive regulators of phagocytosis in iMGL 2.9 compared to 2.0. Red bars represent genes that were significantly different (adjusted *p* < 0.05) between iMGL 2.0 and 2.9 whereas black bars represent genes that were not significantly different. *n* = 4 lines. **B**-**D** iMGL 2.0, iMGL 2.9 and primary human microglia were treated side-by-side with vehicle or pHrodo™ Green-labelled myelin, α-synuclein preformed fibrils (α-syn PFF), opsonized red blood cells (IgG-RBC) or *E. coli* for three hours and then counterstained with Hoechst 33342. **B** Representative fluorescence images. Scale bar = 50 $\mu m$. **C**-**D** Quantification of mean green fluorescence intensity (MFI) per cell. Mann–Whitney tests were performed in C. *n* = 4 lines, * *p* < 0.05 in C. Average values from *n* = 2 lines (iMGL) or donors (primary microglia) are presented in D

*MERTK*, *GAS6* and *PROS1*, which were found to be more highly expressed in iMGL 2.9 compared to iMGL 2.0, are all well known for their involvement in phagocytic processes [15, 27, 43]. In addition, DEG analysis revealed a number of other genes implicated in phagocytosis, such as genes encoding Fcγ receptors, to be more highly expressed in iMGL 2.9 compared to iMGL 2.0 (Fig. 2A). Consistently, phagocytosis assay revealed a higher uptake of pHrodo™ Green-labelled myelin and α-synuclein fibrils (both mediated by MerTK [27, 44]), and IgG-opsonized red blood cells (mediated by Fcγ receptors) by iMGL 2.9 compared to iMGL 2.0 (Fig. 2B-C). No difference in the extent of *E. coli* uptake was observed between iMGL 2.0 and 2.9 (Fig. 2B-C). All substrates were internalized by iMGL 2.9 at a comparable level to primary microglia (Fig. 2B-C). Overall, iMGL generated using the 2.9 protocol are more efficiently able to internalize a variety of substrates than cells generated using the original 2.0 protocol.

### LPS elicits a functional TLR4 response from iMGL 2.9 but not iMGL 2.0

**Fig. 3:**
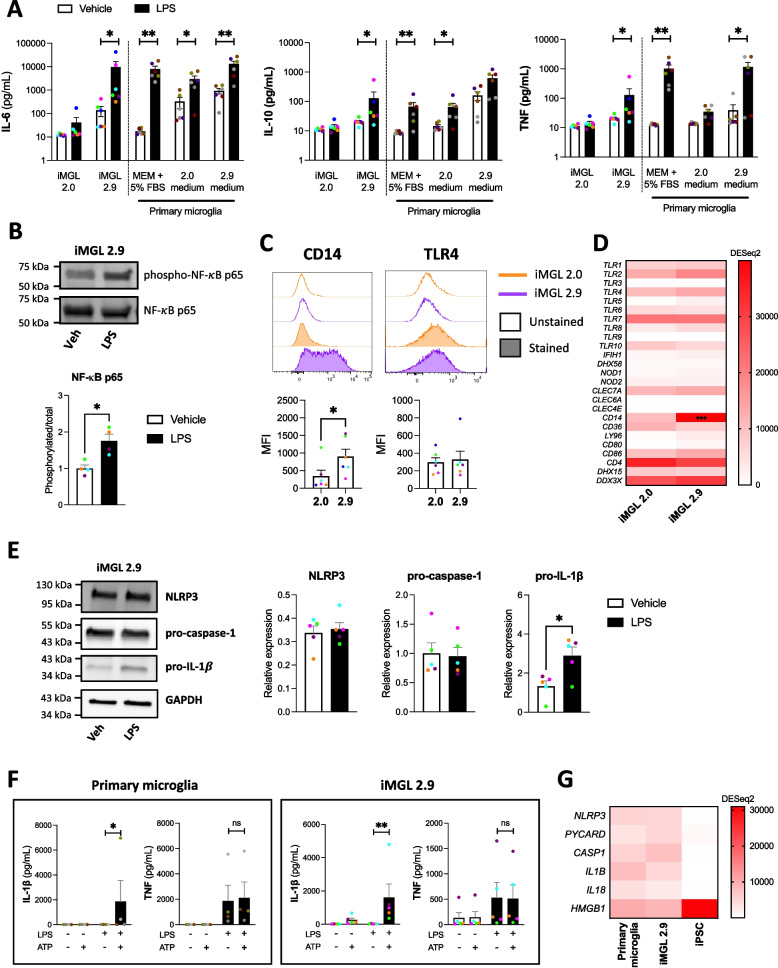
TLR4 signaling response of iMGL 2.9 following LPS treatment. iMGL 2.0 and 2.9 were differentiated side-by-side from the same healthy control iPSC lines. **A** IL-6, TNF and IL-10 concentrations in supernatants from iMGL 2.0, iMGL 2.9 and primary human microglia treated with vehicle or LPS (100 ng/mL) for 24 h. Mann–Whitney tests were performed. *n* = 5 lines for iMGL, *n* = 6 donors of primary microglia, * *p* < 0.05, ** *p* < 0.01, *** *p* < 0.001. **B** Representative Western blotting and quantification of NF-κB in iMGL 2.9 treated with vehicle or LPS (100 ng/mL) for 1.5 h. A Mann–Whitney test was performed. *n* = 4 lines, * *p* < 0.05. **C** Flow cytometry assessment of cell surface CD14 and TLR4 expression in iMGL 2.0 and 2.9. Mann–Whitney tests were performed. n = 6 lines, * *p* < 0.05. **D** Heatmap showing expression of genes encoding pattern recognition receptor and their co-receptors. A two-way ANOVA was performed. *n* = 4 lines, *** *p* < 0.001 vs iMGL 2.0. **E** Representative Western blotting and quantification of inflammasome components in iMGL 2.9 treated with vehicle or LPS (100 ng/mL) for 24 h. Mann–Whitney tests were performed. *n* = 5 lines, * *p* < 0.05. **F** IL-1β and TNF concentrations in cell supernatants of human primary microglia and iMGL 2.9 treated with vehicle or LPS (100 ng/mL) for 24 h, followed by ATP (5 mM) or not for 30 min. Kruskal–Wallis tests followed by Dunn’s multiple comparison tests were performed. *n* = 4 donors of primary microglia and n = 5 lines for iMGL, * *p* < 0.05, ** *p* < 0.01, ns = non-significant. **G** Heatmap showing the expression of genes encoding NLRP3 inflammasome components and their substrates. *n* = 4 donors of primary microglia, 4 lines for iMGL and iPSCs

A major issue previously noted with iMGL differentiated using the 2.0 protocol is their poor inflammatory response to LPS [15, 45], a widely used inflammatory stimulus that agonizes TLR4. Measurement of cytokine secretion following LPS treatment revealed iMGL 2.9 and primary microglia, but not iMGL 2.0, to secrete significantly higher amounts of IL-6, TNF and IL-10 (Fig. 3A). This was associated with increased phosphorylation of the transcription factor NF-κB regulating cytokine expression following LPS treatment (Fig. 3B). Accordingly, flow cytometry (Fig. 3C) and qRT-PCR (Additional file 1: Figure S15) assessment of TLR4 and its co-receptor CD14, essential for LPS recognition [46], revealed CD14 expression to be significantly higher in iMGL 2.9 compared to iMGL 2.0. Expression of lymphocyte antigen 96 (*LY96*), encoding the co-receptor of TLR4 MD2 also essential for LPS recognition [46], was also more highly expressed in iMGL 2.9 compared to iMGL 2.0 as quantified by qRT-PCR (Additional file 1: Figure S15). MD2 cell surface expression was not investigated due to the unavailability of flow cytometry-validated antibodies. RNA-sequencing analysis revealed no difference in the expression of pattern recognition receptors and their co-receptors between iMGL 2.0 and 2.9, except for *CD14* (Fig. 3D). Interestingly, substitution of DMEM/F12 by MEMα in the 2.0 microglia differentiation medium was sufficient to increase the inflammatory response of iMGL to LPS, with addition of GM-CSF having negligible effect (Additional file 1: Figure S16). iMGL 2.9 also showed functional inflammatory response to agonists of other TLRs such as Pam_3_CSK_4_ or R-FSL-1. (Additional file 1: Figure S17A). Secretion of a wide array of chemokines by iMGL 2.9 was observed following LPS or IFNγ treatment, albeit with some discrepancy in secretory pattern compared to primary microglia (Additional file 1: Figure S17B).

The inflammasome is a stimulus-induced multiprotein complex of the innate immune system that have been linked to a variety of diseases, including neurodegenerative diseases [47–50]. iMGL 2.9 had a functional inflammasome system, as evidenced by increased IL-1β, but not TNF release upon ATP treatment, when cells were primed with LPS to increase IL-1β protein expression (Fig. 3E-F). Similarly, cultured primary microglia also showed enhanced IL-1β secretion following LPS priming and ATP induction of the inflammasome (Fig. 3F). iMGL 2.9 and primary microglia in culture had similar basal expression of genes encoding NLRP3 inflammasome components and their substrates, and distinctively differed from iPSCs (Fig. 3G). Overall, our findings imply that iMGL derived using the 2.9 protocol are better suited for studying microglial inflammatory activities compared to those derived using the 2.0 protocol.

### *The c.2350G* > *A (p.V784M) CSF1R variant causes ALSP*

**Fig. 4:**
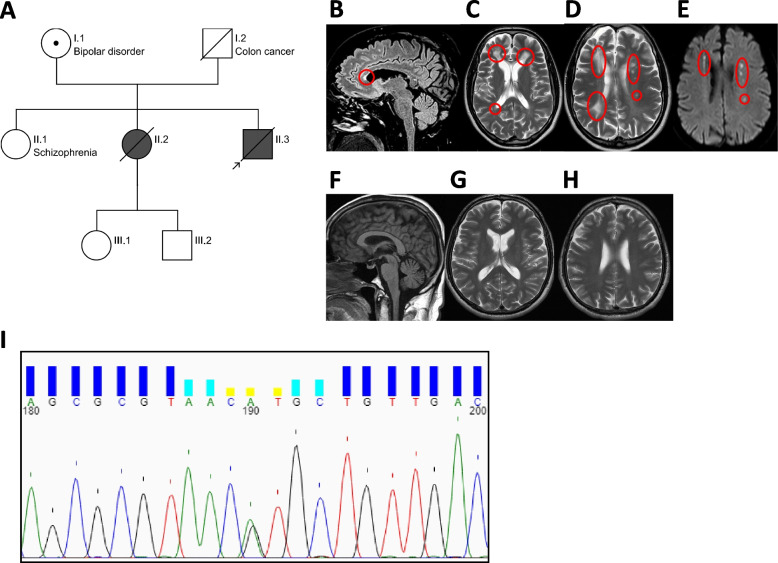
Pedigree of the proband’s family, MRI images, and genotyping data. **A** Pedigree of the proband’s family (prepared via https://cegat.com/). Shading indicates carriers of the c.2350G > A (p.V784M) *CSF1R* variant with ALSP diagnosis. Symbol with a dot indicates a carrier without clinical manifestations of ALSP. **B**-**E** Brain MRI of subject II.3. **B** Sagittal FLAIR T2-weighted MR image showing thinning and hyperintense signal of the anterior portion of the body of the corpus callosum (red circle). **C**-**D** Axial T2-weighted MR images showing the presence of bilateral multifocal and confluent lesions in the frontal lobes (red circles) and multifocal lesions in the left fontal and parietal lobe with areas of restricted diffusion in the diffusion weighted image (**E**, red circles). **F**–**H** Brain MRI of a healthy control, matched by sex and age to subject II.3. **F** Sagittal T1-weighted image showing normal size and signal of the brain parenchyma, specifically of the corpus callosum. **G**-**H** Axial T2-weighted images showing no atrophy, normal ventricular size and normal signal at the level of the cerebral white matter. (I) Chromatogram of PBMC-derived DNA showing heterozygous c.2350G > A *CSF1R* variant (shown here as position 190)

The primary objective of the current study was to develop an in vitro tool to study *CSF1R* variants associated with ALSP in human microglia. A 54-year-old male patient of Croatian origin (Fig. 4A subject II.3) was diagnosed with ALSP in 2020. A missense mutation (c.2350G > A) resulting in the substitution of a highly conserved valine residue by a methionine residue in the tyrosine kinase domain of CSF1R (p.V784M) had been previously described in his deceased sister (Fig. 4A subject II.2) who had also been clinically diagnosed with ALSP [51]. His mother (Fig. 4A subject I.1) is also a carrier of the same *CSF1R* variant and presented with a well-controlled bipolar disease, but no neurological symptoms or dementia. His family history was also positive for schizophrenia in the oldest sister (Fig. 4A subject II.1), who refused genetic testing. A few months prior to the diagnosis, the patient manifested mood swings, personality changes, and trouble performing common tasks at work. Upon assessment at the Montreal Neurological Institute-Hospital, his neurological examination revealed anxiety, mild dysmetria and gait apraxia. Brain MRI documented extensive bilateral white matter abnormalities predominant in the frontal and parietal lobes, more prominent on the right hemispheres (Fig. 4B-D; Fig. 4F-H show brain MRI of an age- and sex-matched healthy control). The corpus callosum was also affected at the level of the genu and anterior portion of the body (Fig. 4B). On diffusion-weighted images, few foci of diffusion restriction in the corona radiata and centrum semiovale were detected bilaterally (Fig. 4E). Genetic sequencing of *CSF1R* documented the presence of the heterozygous c.2350G > A pathogenic variant in the patient, confirming the diagnosis of ALSP (Fig. 4I). Given the clear association of this c.2350G > A (p.784 M) variant with clinical diagnosis of ALSP, the patient’s PBMCs were collected in order to generate iMGL. The patient requested and received medical assistance in dying at the age of 57.

### The 2.9, but not the 2.0 protocol, allows the generation of viable iMGL from an ALSP patient harboring a heterozygous CSF1R pathogenic variant

**Fig. 5:**
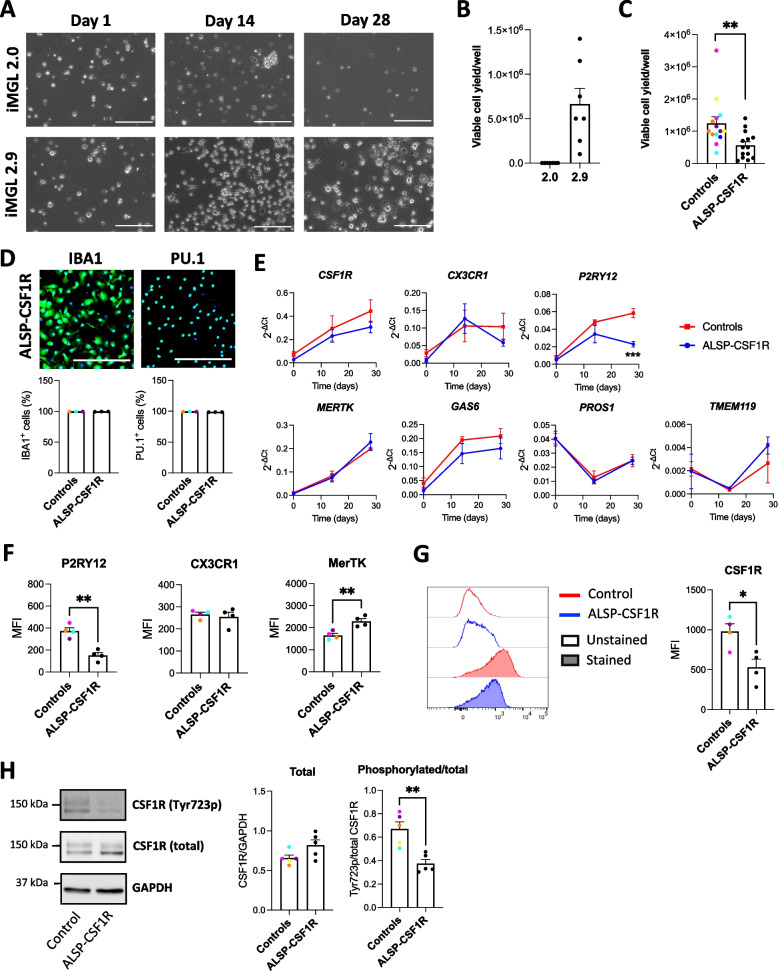
Derivation of microglia-like cells from the ALSP-CSF1R patient with a c.2350G > A (p.V784M) *CSF1R* variant. **A** Phase contrast images of ALSP-CSF1R iPSC line differentiated into iMGL using the 2.0 or 2.9 protocol. Scale bar = 150 $\mu m$. **B** Viable cell yield per well of a 6-well plate assessed by trypan blue exclusion assay following 2.0 and 2.9 differentiation of the ALSP-CSF1R iPSCs into iMGL. *n* = 7 differentiation batches. **C** Viable cell yield per well of a 6-well plate assessed by trypan blue exclusion assay following side-by-side differentiation of healthy control lines (*n* = 14 differentiation batches from 6 lines) and the ALSP-CSF1R line (*n* = 14 differentiation batches) using the 2.9 protocol. A t-test was performed, * *p* < 0.05. (D) Quantification of IBA1- and PU.1-immunopositivity. *n* = 4 healthy control lines and 4 batches of a single ALSP-CSF1R line, differentiated side-by-side using the 2.9 protocol. **E** qRT-PCR assessment of microglia marker expression on day 0, 14 and 28 of microglial differentiation. *n* = 4 healthy control lines and 4 batches of a single ALSP-CSF1R line, differentiated side-by-side using the 2.9 protocol. Two-way ANOVA were performed, followed by Sidak’s post hoc tests. *** *p* < 0.001. **F** Flow cytometry assessment of P2RY12, CX3CR1 and MerTK cell surface expression. T-tests were performed. *n* = 4 healthy control lines and 4 batches of a single ALSP-CSF1R line, differentiated side-by-side using the 2.9 protocol. ** *p* < 0.01. **G** Flow cytometry assessment of CSF1R cell surface expression. A t-test was performed. *n* = 4 healthy control lines and 4 batches of a single ALSP-CSF1R line, differentiated side-by-side using the 2.9 protocol. * *p* < 0.05. **H** Western blot assessment of CSF1R and its tyrosine 723-phosphorylated form, and GAPDH. A t-test was performed. *n* = 5 healthy control lines and 5 batches of a single ALSP-CSF1R line, differentiated side-by-side using the 2.9 protocol. ** *p* < 0.01

Patient’s PBMCs were first reprogrammed into iPSCs that expressed the pluripotency markers Nanog, Tra-1–60, SSEA-4 and OCT3/4 (Additional file 2: Figure S18A). Presence of a c.2350G > A variant in the *CSF1R* gene was confirmed by Sanger sequencing (Additional file 2: Figure S18B). Karyotyping and qPCR-based screening did not reveal any genomic abnormality (Additional file 2: Figure S18C-D). The patient line will be referred to as “ALSP-CSF1R”. Differentiation of ALSP-CSF1R iPSCs into iHPCs resulted in round floating CD43 + cells, with a fraction also expressing CD34 (Additional file 2: Figure S19A-B) as expected [4]. While the 2.0 protocol failed to generate any viable microglial cells from the ALSP-CSF1R iHPCs, the 2.9 protocol successfully induced their microglial differentiation (Fig. 5A-B). Interestingly, addition of GM-CSF to the 2.0 microglia differentiation medium and the substitution of DMEM/F12 with MEMα, but neither of these medium modifications alone, resulted in the robust generation of viable iMGL from ALSP-CSF1R iHPCs (Additional file 2: Figure S20). When the 2.9 protocol was used, the final cell yield was significantly lower with the ALSP-CSF1R line compared to healthy control lines differentiated side-by-side (Fig. 5C). Ki67 immunostaining and PI staining did not reveal significant difference in proliferation and death rate, respectively, in mature iMGL culture between control and ALSP-CSF1R (Additional file 2: Figure S21A-D). ~ 100% and ~ 99% of the ALSP-CSF1R iMGL expressed PU.1 and IBA1, respectively (Fig. 5D), indicative of successful myeloid differentiation. The cells showed a time-dependent increase in the expression of microglia marker genes such as *CX3CR1*, *GAS6* and transmembrane protein 119 (*TMEM119*) throughout their differentiation, however *P2RY12* expression was observed to decline over the latter half of the differentiation period (Fig. 5E). This resulted in significantly lower expression of *P2RY12* in ALSP-CSF1R iMGL compared to healthy controls (Fig. 5E). Flow cytometry assessment revealed lower P2RY12 cell surface expression (Fig. 5F), consistent with lower *P2RY12* mRNA expression (Fig. 5E), in ALSP-CSF1R iMGL compared to controls. Cell surface expression of CX3CR1 and MerTK was respectively no different and higher in ALSP-CSF1R iMGL compared to controls (Fig. 5F).

ALSP-CSF1R iMGL expressed the c.2350G > A *CSF1R* variant, whereas none of the iMGL used as controls had pathogenic variants in the region encoding CSF1R tyrosine kinase domain (Additional file 5: Table S3). *CSF1R* mRNA (Fig. 5E) and protein (Fig. 5H) expression in ALSP-CSF1R iMGL was no different from healthy controls, yet cell surface expression of CSF1R measured by flow cytometry was significantly lower (Fig. 5G), indicative of a defective cell surface trafficking or recycling of CSF1R in the patient-derived cells. This was consistent with a previous report that ALSP-associated mutant CSF1R accumulate in Golgi-like perinuclear regions, resulting in reduced cell surface expression [52]. Assessment of CSF1R phosphorylation at tyrosine 723 revealed lower activation of CSF1R in ALSP-CSF1R iMGL compared to controls (Fig. 5H), in line with previous observations that ALSP-associated variants in *CSF1R* impairs M-CSF-induced autophosphorylation [53, 54].

Overall, yield assessment and molecular characterization revealed that ALSP-CSF1R iPSCs could be successfully differentiated into iMGL through the 2.9 protocol, but not the original 2.0 protocol.

### ALSP-CSF1R iMGL presents phenotypic alterations compared to healthy control iMGL

**Fig. 6:**
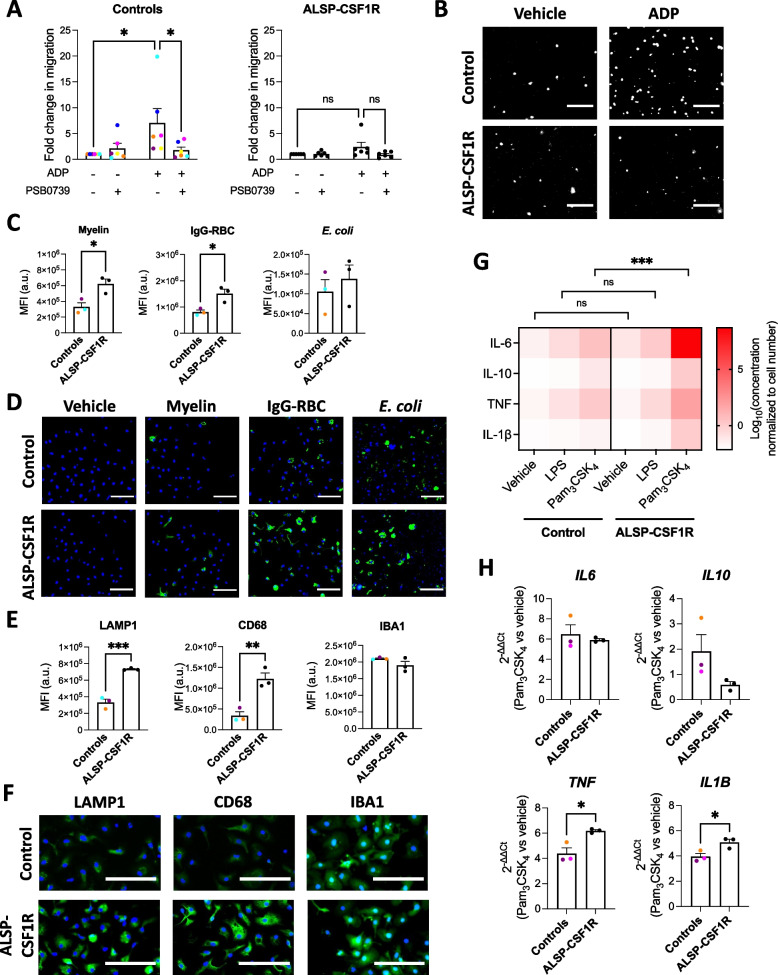
Functional phenotype of iMGL derived from the ALSP-CSF1R patient with a c.2350G > A (p.V784M) *CSF1R* variant. All iMGL were generated using the 2.9 protocol. Quantification (**A**) and images (**B**) of iMGL migratory activity toward ADP assessed by Boyden chamber assay. Cells were concomitantly treated or not with PSB0739. Kruskal–Wallis tests followed by Dunn’s multiple comparison tests were performed. *n* = 6 healthy control lines and 6 batches of a single ALSP patient line, differentiated side-by-side. White = Hoechst 33342, scale bar = 75 μm in (**B**). Quantification of green fluorescence intensity per cell (**C**) and representative images (**D**) of iMGL exposed to vehicle or pHrodo.™ Green-labelled myelin, opsonized red blood cells (IgG-RBC) or *E. coli* for three hours and then counterstained with Hoescht 33342. T-tests were performed. n = 3 healthy control lines and 3 batches of a single ALSP patient line, differentiated side-by-side, * *p* < 0.05. Scale bar = 250 $\mu m$ in (**D**). Quantification of mean fluorescence intensities (MFI; **E**) and representative images (**F**) of LAMP1, CD68 and IBA1 immunostaining of iMGL. T-tests were performed. *n* = 3 healthy control lines and 3 batches of a single ALSP patient line, differentiated side-by-side, ** *p* < 0.01, *** *p* < 0.001. Scale bar = 100 $\mu m$ in (**F**). **G** Cytokine secretion assessed in cell supernatants following a 24-h treatment with vehicle, LPS (100 ng/mL) or Pam_3_CSK_4_ (100 ng/mL). A two-way ANOVA was performed, followed by Tukey’s post hoc test. *n* = 4 healthy control lines and 4 batches of a single ALSP patient line, differentiated side-by-side, *** *p* < 0.001, ns = non-significant. **H** qRT-PCR performed after three hours of Pam_3_CSK_4_ (100 ng/mL) vs. vehicle treatment. T-tests were performed. *n* = 3 healthy control lines and 3 batches of a single ALSP patient line, differentiated side-by-side, * *p* < 0.05

P2RY12 is a purinergic receptor important for chemotactic migration of microglia toward nucleotides released by dead cells [55]. Since *P2RY12* expression was observed to be lower in ALSP-CSF1R iMGL compared to controls, migratory activity was assessed using a Boyden chamber assay. While healthy control iMGL showed significant migration toward ADP (~ sevenfold increase in migrating cells, *p* = 0.0103) that was blocked by the P2RY12 antagonist PSB0739, ALSP-CSF1R iMGL showed minimal migration toward ADP (~ twofold increase in migrating cells, *p* = 0.0876; Fig. 6A-B).

The phagocytosis receptor MerTK was observed to be more highly expressed in ALSP-CSF1R iMGL compared to controls. Phagocytosis assays using pHrodo™ Green-labelled substrates revealed a higher uptake of myelin and IgG-opsonized red blood cells by ALSP-CSF1R iMGL compared to healthy controls (Fig. 6C-D). Slightly higher uptake of *E. coli* was also observed in patient-derived cells, although this did not reach statistical significance (Fig. 6C-D). Time course experiments using pHrodo™ Green-labelled myelin pointed toward enhanced uptake capacity, rather than enhanced uptake rate, of the ALSP-CSF1R iMGL compared to controls (Additional file 2: Figure S22A). pHrodo™ Green is a pH-sensitive dye that allows the selective quantification of labelled targets that reaches acidic compartments of the cells upon phagocytosis [56, 57]. Uptake assay using FITC-labelled myelin resulted in similar observations as with pHrodo™ Green-labelled myelin (Additional file 2: Figure S22B), excluding lysosomal pH as a factor influencing phagocytosis assay results. In line with this, lysosomal pH assessment using the ratiometric LysoSensor™ Yellow/Blue probe revealed no difference in lysosomal pH between control and ALSP-CSF1R iMGL (Additional file 2: Figure S23A; ammonium chloride was used as a control to increase lysosomal pH). Immunostaining intensities of the lysosomal markers LAMP1 and CD68 were higher in ALSP-CSF1R iMGL compared to healthy controls (Fig. 6E-F). In contrast, staining intensities of the calcium-binding protein IBA1 were found to be similar between ALSP-CSF1R and control cells (Fig. 6E-F). Overall, these data suggest that lysosomal content is higher in ALSP-CSF1R iMGL compared to controls, which could account for a higher phagocytic capacity of the patient-derived cells. DQ™ red BSA is a labelled BSA that emits fluorescence upon BSA proteolysis in lysosomes. No difference in DQ™ red BSA proteolysis was observed between control and ALSP-CSF1R iMGL over the course of 24 h (Additional file 2: Figure S23B-C; the lysosomal acidification inhibitor bafilomycin A1 was used as a control to inhibit DQ™ red BSA proteolysis). This indicates that despite higher internalization and lysosomal storage of phagocytosed materials, lysosomal protease activities are not higher in ALSP-CSF1R iMGL compared to controls.

Lastly, the inflammatory response of ALSP-CSF1R iMGL to LPS and Pam_3_CSK_4_ was compared to that of control iMGL. Higher cytokine secretion upon 24-h Pam_3_CSK_4_, treatment was observed in ALSP-CSF1R iMGL culture compared to control iMGL culture (Fig. 6G). This was corroborated by a greater upregulation of *TNF* and *IL1B* mRNA following a 3-h Pam_3_CSK_4_ treatment of ALSP-CSF1R iMGL compared to control iMGL (Fig. 6H). No significant difference in *IL6* and *IL10* upregulation was observed between Pam_3_CSK_4_-treated ALSP-CSF1R iMGL compared to controls at the studied timepoint (Fig. 6H). ALSP-CSF1R iMGL also appeared to have a heightened response to LPS, but this did not reach statistical significance.

In sum, these findings suggest that the heterozygous c.2350G > A (p.V784M) *CSF1R* variant might be associated with phenotypic alterations of microglia including reduced chemotaxis toward ADP, higher phagocytic capacity owing to a larger lysosomal storage capacity, and heightened TLR-mediated inflammatory response.

### CSF1R loss of function results in P2RY12 downregulation

In order to confirm that phenotypic and functional alterations observed in ALSP-CSF1R iMGL are due to *CSF1R* haploinsufficiency, iMGL were additionally generated from iPSC cell lines with CRISPR-Cas9 edition of the *CSF1R* gene.

**Fig. 7:**
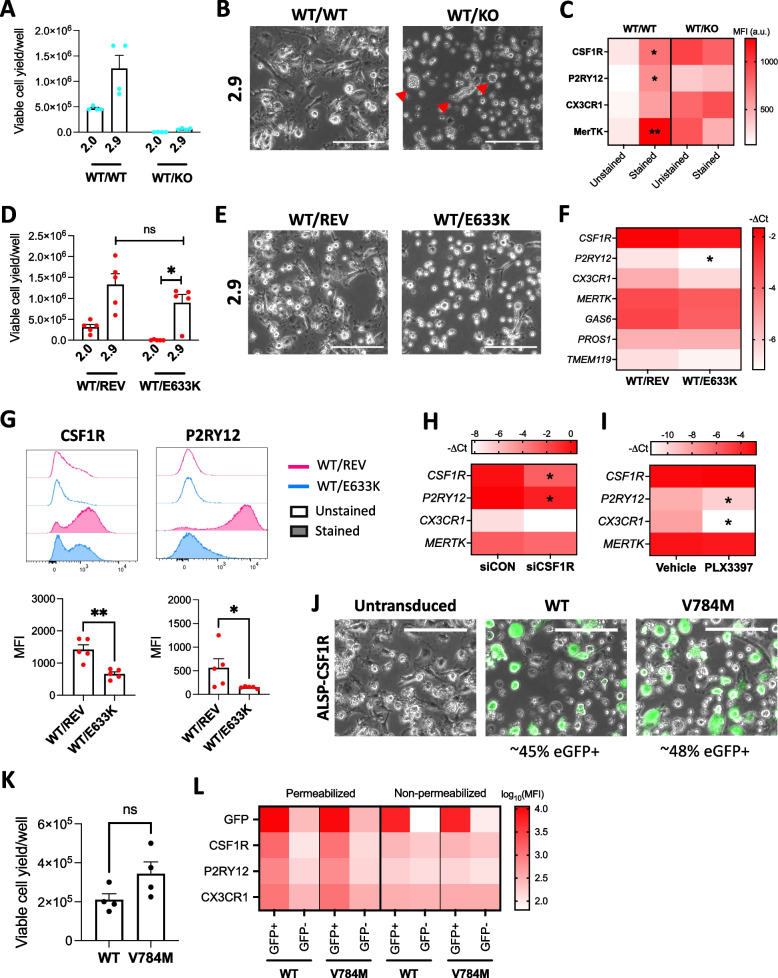
Effect of CSF1R loss of function on microglial phenotype. **A**-**C** *CSF1R*^*WT/WT*^ and *CSF1R*^*WT/KO*^ iPSCs were differentiated into iMGL side-by-side. **A** Viable cell yield per well of a 6-well plate assessed by trypan blue exclusion assay following 2.0 and 2.9 differentiation. A Kruskal–Wallis test was performed, followed by Dunn’s post hoc test. *n* = 4 differentiation batches. **B** Phase contrast images of iMGL 2.9. Red triangles show presence of round or dysmorphic floating cells. Scale bar = 150 $\mu m$. **C** Flow cytometry assessment of cell surface marker expression. Kruskal–Wallis tests were performed, followed by Dunn’s post hoc test. *n* = 4 differentiation batches using the 2.9 protocol, * *p* < 0.05, ** *p* < 0.01 vs. unstained control. MFI = median fluorescence intensity, a.u. = arbitrary unit. **D**-**G** *CSF1R*^*WT/REV*^ and *CSF1R*^*WT/E633K*^ iPSCs were differentiated into iMGL side-by-side. **D** Viable cell yield per well of a 6-well plate assessed by trypan blue exclusion assay following 2.0 and 2.9 differentiation. A Kruskal–Wallis test was performed, followed by Dunn’s post hoc test. *n* = 4 differentiation batches. **E** Phase contrast images of iMGL 2.9. Scale bar = 150 $\mu m$. **F** qRT-PCR assessment of microglia markers. T-tests were performed. *n* = 5 differentiation batches using the 2.9 protocol, * *p* < 0.05 vs *CSF1R*^*WT/REV*^ iMGL. **G** Flow cytometry assessment of cell surface marker expression. Mann–Whitney tests were performed. n = 5 differentiation batches using the 2.9 protocol * *p* < 0.05, ** *p* < 0.01. MFI = median fluorescence intensity. **H**-**I** Healthy control, mature iMGL were generated following the 2.9 protocol. **H** Microglia marker expression assessed by qRT-PCR in iMGL three days after siCON or siCSF1R transfection. T-tests were performed. *n* = 3 lines, * *p* < 0.05 vs siCON. **I** Microglia marker expression assessed by qRT-PCR in iMGL following a 3-day treatment with PLX3397 (1 μM, every other day). T-tests were performed. *n* = 4 lines, * *p* < 0.05 vs vehicle treatment. **J**-**L** eGFP and either WT or V784M CSF1R were stably co-expressed in ALSP-CSF1R iHPCs using lentiviruses and cells were differentiated into iMGL following the 2.9 protocol. **J** Merged phase contrast and green fluorescence images of ALSP-CSF1R iMGL. Scale bar = 150 μm. (K) Viable cell yield per well of a 6-well plate assessed by trypan blue exclusion assay. A t-test was performed. *n* = 4 differentiation batches. **L** Flow cytometry assessment of eGFP signal and microglia marker expression. MFI = median fluorescence intensity. *n* = 1 differentiation batch

A 4-base pair deletion in *CSF1R* (c.2330_2333del) resulted in a frameshift and a subsequent premature stop codon formation (predicted protein sequence changes = p.R777PfsX9; Additional file 2: Figure S24A-B). A ~ 53% decrease in transcriptional expression of *CSF1R* was observed in this CRISPR-Cas9-edited iPSC line compared to its non-edited isogenic control line (Additional file 2: Figure S24C), suggestive of nonsense-mediated decay of the transcript containing the premature stop codon [58]. The edited iPSCs expressed pluripotency markers (Additional file 2: Figure S24D), and karyotyping and qPCR-based screening did not reveal any genomic abnormality (Additional file 2: Figure S24E-F). The edited cell line and its isogenic control line will be henceforth referred to as “*CSF1R*^*WT/KO*^” and “*CSF1R*^*WT/WT*^”, respectively. Interestingly, a ~ 93% decrease in transcriptional expression of *CSF1R* was observed in *CSF1R*^*WT/KO*^ compared to *CSF1R*^*WT/WT*^ iHPCs (Additional file 2: Figure S24C). Differentiation of the *CSF1R*^*WT/KO*^ line into iMGL following the 2.9 protocol resulted in significantly poorer yield compared to the *CSF1R*^*WT/WT*^ line (Fig. 7A; ~ 0.06 × 10^6^/well compared to ~ 1.25 × 10^6^/well). Viable iMGL could be generated from the *CSF1R*^*WT/WT*^, but not the *CSF1R*^*WT/KO*^ line using the 2.0 protocol (Fig. 7A). Microscopic observation revealed dead cells and cellular debris in the *CSF1R*^*WT/KO*^ iMGL 2.9 culture, with occasional presence of floating round or dysmorphic cells that lacked typical microglial morphology (Fig. 7B). Flow cytometry assessment of cell surface microglia marker expression revealed high autofluorescence at any channels tested, and lack of CSF1R, P2RY12, CX3CR1 and MerTK expression (Fig. 7C). CSF1R, P2RY12, CX3CR1 and MerTK cell surface expression was detectable on *CSF1R*^*WT/WT*^ iMGL (Fig. 7C). Overall, data suggest CSF1R loss of function impairs microglial differentiation.

Microglial differentiation of a commercially available iPSC line expressing a CRISPR-Cas9-induced ALSP-associated c.1897G > A (p.E633K) *CSF1R* variant revealed viable iMGL production using the 2.9, but not the 2.0 protocol (Fig. 7D). Viable iMGL could be generated using either protocol from the isogenic control line in which the c.1897G > A mutation was reverted by CRISPR-Cas9 editing (“*CSF1R*^*WT/REV*^”). Curiously, no significant difference in viable iMGL yield was observed between the *CSF1R*^*WT/E633K*^ and the *CSF1R*^*WT/REV*^ line when the 2.9 protocol was employed (Fig. 7D-E). The p.E633K CSF1R variant was associated with decreased phosphorylation ratio (Additional file 2: Figure S25A; in line with a previous observation [53]) and cell surface expression of CSF1R despite similar transcriptional expression of *CSF1R* between *CSF1R*^*WT/E633K*^ and *CSF1R*^*WT/REV*^ iMGL (Fig. 7F-G). Among all tested microglia markers, only *P2RY12* mRNA expression was found to be significantly lower in *CSF1R*^*WT/E633K*^ iMGL compared to *CSF1R*^*WT/REV*^ iMGL (Fig. 7F). Lower expression of cell surface P2RY12 in *CSF1R*^*WT/E633K*^ iMGL was confirmed by flow cytometry (Fig. 7G). This is consistent with the poor P2RY12 expression observed in ALSP-CSF1R iMGL with a p.V784M CSF1R variant. Myelin uptake level was similar between *CSF1R*^*WT/E633K*^ and *CSF1R*^*WT/REV*^ iMGL (Additional file 2: Figure S25B-C), whereas the level of basal and Pam_3_CSK_4_-induced cytokine secretion was lower in *CSF1R*^*WT/E633K*^ iMGL compared to *CSF1R*^*WT/E633K*^ iMGL (Additional file 2: Figure S25D). These were inconsistent with the heightened myelin uptake and inflammatory response observed in ALSP-CSF1R iMGL compared to healthy controls.

Next, CSF1R function was acutely abrogated in healthy control, mature iMGL through siRNA-mediated knockdown or pharmacological inhibition. *CSF1R* knockdown resulted in significantly decreased *CSF1R, P2RY12* and *CX3CR1*, but not *MERTK* expression after three days (Fig. 7H, Additional file 2: Figure S26A). Cell viability was not compromised at this time point (Additional file 2: Figure S26B). No change in *CSF1R* and *MERTK* and a significant decrease in *P2RY12* and *CX3CR1* expression were observed after three days of treatment with the CSF1R inhibitor PLX3397 (Fig. 7I, Additional file 2: Figure S26C). PLX3397 at the employed concentration induced a decrease in iMGL viability after six days, but not after 3 days of treatment (Additional file 2: Figure S26D). Neither CSF1R knockdown nor inhibition were found to impact myelin uptake (Additional file 1: Figure S26E-F) and Pam_3_CSK_4_-induced cytokine secretion (Additional file 2: Figure S26G-H). These suggest acute loss in CSF1R function is sufficient to cause *P2RY12* downregulation in mature iMGL.

Finally, ALSP-CSF1R iHPCs heterozygous for the c.2350G > A (p.V784M) *CSF1R* variant were transduced with lentiviruses to stably co-express eGFP and either WT or p.V784M CSF1R. Upon microglial differentiation, respectively ~ 45% and ~ 48% of the culture exposed to WT and p.V784M CSF1R lentiviruses were found to be eGFP + (Fig. 7J, Additional file 2: Figure S27). Curiously, no difference in iMGL 2.9 yield was observed between cultures transduced with WT and p.V784M CSF1R lentiviruses. In WT *CSF1R*-transduced culture, the expression of CSF1R, P2RY12 and CX3CR1 was higher in eGFP + compared to eGFP- cells, suggesting functional CSF1R signaling promotes the acquisition of P2RY12 and CX3CR1 expression (Fig. 7L). Similar observations were made in p.V784M *CSF1R*-transduced culture, suggesting the p.V784M CSF1R variant has a partial loss of function that can be circumvented by its overexpression (Fig. 7L). This is in line with the lack of difference in iMGL yield between cultures transduced with WT and p.V784M *CSF1R* lentiviruses. Higher myelin uptake was observed in eGFP + compared to eGFP- cells in both WT and p.V784M *CSF1R*-transduced cultures (Additional file 2: Figure S28A-B), suggesting CSF1R expression during microglial differentiation promotes myelin uptake ability.

Overall, data suggest that i) our 2.9 protocol allows the study of iMGL bearing ALSP-associated pathogenic *CSF1R* variants, and ii) poor P2RY12 expression observed in ALSP-CSF1R iMGL is due to *CSF1R* haploinsufficiency.

## Discussion

Limited access to primary human microglia has long hampered advances in our understanding of microglia biology and their role in human diseases. This is especially true for rare diseases such as ALSP. iMGL technology offers the possibility to study phenotypic and functional alterations of microglia in the context of such rare diseases, and potentially to understand patient-specific defects. It is imperative that successful iMGL modelling of ALSP is achieved in order to develop and assess the effectiveness of therapies.

We have developed a new protocol of microglia derivation from iPSCs, resulting in a significant improvement in cell yield, adhesive properties, protein expression of select microglia markers, scavenging activities and inflammatory response compared to the original protocol published by McQuade et al*.* in 2018. Transcriptomic profile of the resulting iMGL also more closely resembled that of ex vivo and in vitro primary human microglia. This new protocol was applied to successfully generate iMGL from a *CSF1R*-mutated ALSP patient. Characterization of the ALSP-CSF1R iMGL carried out in the current study revealed lower CSF1R cell surface expression and autophosphorylation compared to healthy control iMGL, as well as phenotypic alterations affecting P2RY12-mediated migration, myelin uptake and inflammatory response. Poor P2RY12 expression observed in ALSP-CSF1R iMGL compared to healthy control iMGL was confirmed to be a result of *CSF1R* haploinsufficiency.

Postmortem histological studies of ALSP patients have previously uncovered reduced microglia densities (decreased IBA1 + cells [59–61]) and decreased expression of microglia homeostatic markers including P2RY12 compared to control individuals [59]. In contrast, increased number of dysmorphic CD68 + cells have been observed [59, 61]. Those observations pointed toward an altered phenotype of resident microglia and/or migration of peripheral monocytes into the ALSP brain. In the current study, lower cell yield upon microglial differentiation of ALSP-CSF1R and *CSF1R*^*WT/KO*^ iPSCs was observed, consistent with the developmental role of CSF1R and histological observations made in ALSP patients. In addition, significantly lower expression of *P2RY12* and higher expression of CD68 in ALSP-CSF1R iMGL was observed compared to controls. This would suggest that phenotypic alterations of microglia, rather than monocyte migration to the brain, are responsible for the histological observations made in ALSP.

High dependency of lineage commitment on CSF1R signaling has been described for microglia and tissue-resident macrophages but not circulating monocytes [8, 62, 63]. In the brain, CSF1R is almost exclusively expressed by microglia [64, 65]. As such, ALSP is considered a primary microgliopathy. Given the importance of CSF1R signaling throughout microglia development and maintenance, it remains unclear why ALSP typically manifests late in life (43-years-old on average [13]). Aside from CSF1R, another growth factor receptor which promotes microglia proliferation is colony-stimulating factor 2 receptor (CSF2R). Both the CSF1R ligand M-CSF and the CSF2R ligand GM-CSF have been observed to have mitogenic effect on fetal and adult microglia in vitro [66]. GM-CSF and CSF2R expression is detectable in the human brain during fetal development [67]. Yet, whereas *Csf1r* knockout results in an almost complete absence of microglia in mice [8, 12], *Csf2r* knockout does not impact microglia density [68]. These observations suggest that CSF1R but not CSF2R is essential for microglia development. GM-CSF is poorly expressed in the adult brain [67, 68], but is upregulated in some inflammatory contexts such as multiple sclerosis [69, 70], in which it is thought to exert its mitogenic effect on microglia. Interestingly, increased GM-CSF expression has been previously detected in the grey matter of ALSP patients [59, 68]. This was associated with a less pronounced molecular and morphological alterations of microglia in the grey matter compared to the white matter of those patients [59, 61], suggesting CSF2R signaling might play a compensatory role to CSF1R signaling in ALSP patients. Accordingly, addition of GM-CSF to the microglia differentiation medium was essential for the successful derivation of iMGL from ALSP-CSF1R iPSCs.

CSF1R signaling is known to be important for microglial proliferation in response to stress or injury, with increased M-CSF expression observed upon in vivo LPS treatment [71], upon ischemic injury [72], in Alzheimer’s disease and its animal models [71, 73, 74], and in aged mice [71] and human [75]. It can be hypothesized that CSF1R-mediated proliferation or functional changes of microglia are of increased importance in maintaining brain homeostasis during aging, explaining the late-onset of ALSP. In mice, aging has been associated with myelin fragmentation and increased sarkosyl-insoluble lipofuscin accumulation in lysosomes of white matter microglia, suggestive of increased lysosomal burden with aging [76]. The presence of pigmented lipid-laden or lipofuscin-rich CD68 + myeloid cells in the white matter is a pathological hallmark of ALSP [77–81]. Here, ALPS-CSF1R iMGL were observed to have a higher capacity to internalize myelin debris, possibly owing to a higher lysosomal content, compared to control iMGL. Similarly, microglia from *Csf1r*^+/-^ mice have been previously shown to exhibit higher CD68 expression and phagocytosis of myelin compared to microglia from *Csf1r*^+/+^ mice [82]. The excess storage of internalized myelin might be responsible for lipid and lipofuscin accumulation within microglia in the ALSP brain.

The association between ALSP and variants located in the coding region of CSF1R’s kinase domain was established in 2011 [53]. Given the variable clinical expressivity and incomplete penetrance of *CSF1R* variants observed even within family members sharing the same variant [51, 83, 84], it is likely that other genetic, biological or environmental factors contribute to the severity of the resulting phenotype. Generation of an isogenic control iPSC line in the future will be important in isolating microglial dysfunction caused by *CSF1R* pathogenic variant from that caused by other genetic factors in our ALSP-CSF1R line. Generation of iMGL from other ALSP patients will help identify common microglial features specific to ALSP patients. Moreover, unbiased, -omic approaches should allow us to better grasp the full picture of phenotypic alterations present in ALSP microglia. In the current study, poor P2RY12 expression in ALSP-CSF1R iMGL was confirmed to be a consequence of CSF1R loss of function. The greater myelin uptake capacity and inflammatory response of ALSP-CSF1R iMGL are unlikely to result from CSF1R dysregulation, as these traits were not observed in *CSF1R*^*WT/E633K*^ iMGL relative to their isogenic control iMGL. Furthermore, lentiviral transduction of WT CSF1R into ALSP-CSF1R cells resulted in increased myelin uptake.

The newly devised 2.9 protocol for iMGL generation will be useful not only in the context of ALSP, but potentially for the in vitro study of microglia in any neurological diseases. The new protocol conserved the simplicity of the 2.0 protocol developed by McQuade et al., not requiring any cell sorting nor the use of a hypoxia chamber to generate progenitor cells. Composition of the microglia differentiation medium was simplified, requiring less reagents. Generation of iMGL is labor-intensive and involves the use of expensive recombinant proteins. The higher yields achieved by the 2.9 over the 2.0 protocol will allow for a significant cost reduction. In addition, cells differentiated using the 2.9 protocol are more adherent to cultureware, as seen with other previously published iMGL protocols [22–24, 35, 37, 38, 85–87], which is convenient when performing image-based assays. Most importantly, transcriptional profile and functional competence of iMGL 2.9 was substantially closer to that of primary microglia, signifying higher translatability of research findings to human disease. Although studies have validated the inflammatory and/or phagocytic competence of iMGL generated using previously published protocols [22–24, 35, 37, 38, 85, 86], our study represents one of the rare cases in which side-by-side functional comparison of iMGL was made to primary microglia of postnatal sources.

## Conclusions

In conclusion, our newly devised 2.9 protocol for iMGL generation is a promising tool in studying molecular and functional alterations of microglia caused by pathogenic variants associated with human diseases, including primary microgliopathies such as ALSP. This study represents the first successful attempt at investigating iMGL derived from a *CSF1R*-mutated ALSP patient, and revealed molecular alterations that are consistent with previous histopathological findings made in ALSP.

### Supplementary Information

**Supplementary Material 1.**

**Supplementary Material 2.**

**Supplementary Material 3.**

**Supplementary Material 4.**

**Supplementary Material 5.**

## Data Availability

RNA-sequencing data will be made available on Gene Expression Omnibus upon acceptance of this manuscript for publication.

## Abbreviations

ADP: Adenosine diphosphate
ALSP: Adult-onset leukoencephalopathy with axonal spheroids and pigmented glia
APOE: Apolipoprotein E
BSA: Bovine serum albumin
CCL3: Chemokine ligand 3
CD11b: Cluster of differentiation 11b
CD14: Cluster of differentiation 14
CD200: Cluster of differentiation 200
CD34: Cluster of differentiation 34
CD43: Cluster of differentiation 43
CD68: Cluster of differentiation 68
CR1: Complement receptor 1
CSF1R: Colony-stimulating factor 1 receptor
CSF2R: Colony-stimulating factor 2 receptor
CSTB: Cathepsin B
CX3CL1: C-XC-3 motif chemokine ligand 1
CX3CR1: C-XC-3 motif chemokine receptor 1
DMEM: Dulbecco’s Modified Eagle Medium
EDTA: Ethylenediaminetetraacetic acid
FBS: Fetal bovine serum
FITC: Fluorescein isothiocyanate
GAPDH: Glyceraldehyde 3-phosphate dehydrogenase
GAS6: Growth arrest specific 6
GBA: Glucocerebrosidase
GM-CSF: Granulocyte–macrophage colony-stimulating factor
GPR34: G-protein coupled receptor 34
IBA1: Ionized calcium binding adaptor molecule 1
IFNγ: Interferon gamma
iHPC: Induced pluripotent stem cell-derived hematopoietic progenitor cell
IL-10: Interleukin 10
IL-1β: Interleukin 1 beta
IL-34: Interleukin 34
IL-6: Interleukin 6
iMGL: induced pluripotent stem cell-derived microglia
iPSC: Induced pluripotent stem cell
LAMP1: Lysosomal-associated membrane protein 1
LPS: Lipopolysaccharide
LRRK2: Leucine-rich repeat kinase 2
LY96: Lymphocyte antigen 96
M-CSF: Macrophage colony-stimulating factor
MEM: Minimum Essential Medium
MERTK: Mer tyrosine kinase
MMP8: Metalloproteinase 8
mRNA: Messenger ribonucleic acid
NF-κB: Nuclear factor kappa B
NLRP3: NLR family pyrin domain containing 3
OCT-3/4: Octamer binding transcription factor ¾
P2RY12: Purinergic receptor P2Y12
PBMC: Peripheral blood mononuclear cell
PBMC-Mφ: Peripheral blood mononuclear cell-derived macrophages
PCA: Principal component analysis
PROS1: Protein S
P/S: Penicillin/streptomycin
qPCR: Quantitative polymerase chain reaction
qRT-PCR: Real-time quantitative reverse transcription polymerase chain reaction
RNA: Ribonucleic acid
SDS: Sodium dodecyl sulfate
SEM: Standard error of the mean
SIGLEC10: Sialic acid binding Ig-like lectin 10
siRNA: Small interfering RNA
SSEA-4: Stage-specific embryonic antigen-4
TGF-β1: Transforming growth factor beta 1
TLR4: Toll-like receptor 4
TMEM119: Transmembrane protein 119
TNF: Tumor necrosis factor
TREM2: Triggering receptor expressed on myeloid cell 2
YWHAZ: Tyrosine 3-monooxygenase/tryptophan 5-monooxygenase activation protein zeta
