# Supplementary material for: An adapted protocol to derive microglia from stem cells and its application in the study of CSF1R-related disorders
Source: Mol Neurodegener. 2024 Apr 5;19:31. doi: 10.1186/s13024-024-00723-x (PMC10996091; doi:10.1186/s13024-024-00723-x)
Supplement: Supplementary file 1 — Supplementary Material 1. [file 13024_2024_723_MOESM1_ESM.pdf]

A

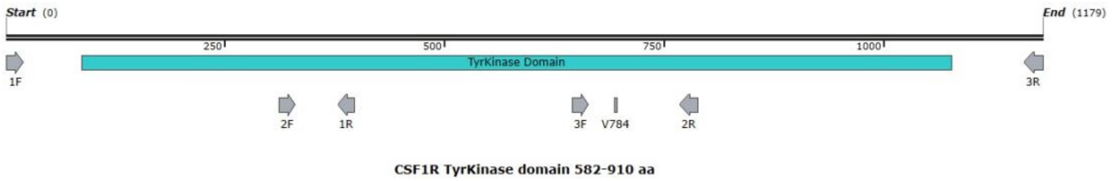

B

Primer name	Sequence (5'-3')
CSF1R-TyrKinase-1F	TCGAGAGCTATGAGGGCAAC
CSF1R-TyrKinase-1R	GTCCCAGCATGGCCTCAG
CSF1R-TyrKinase-2F	AGGCCCTGTACTGGTCATCA
CSF1R-TyrKinase-2R	GCATTGCCCTTGACAATGTA
CSF1R-TyrKinase-3F	TTCCTCGCTTCCAAGAATTG
CSF1R-TyrKinase-3R	TCTCCTCCTCCAGCTCACTG

Figure S1. Sequencing strategy of the region encoding CSF1R tyrosine kinase domain. (A) Locations where the primers would anneal. (B) Sequences of primers.

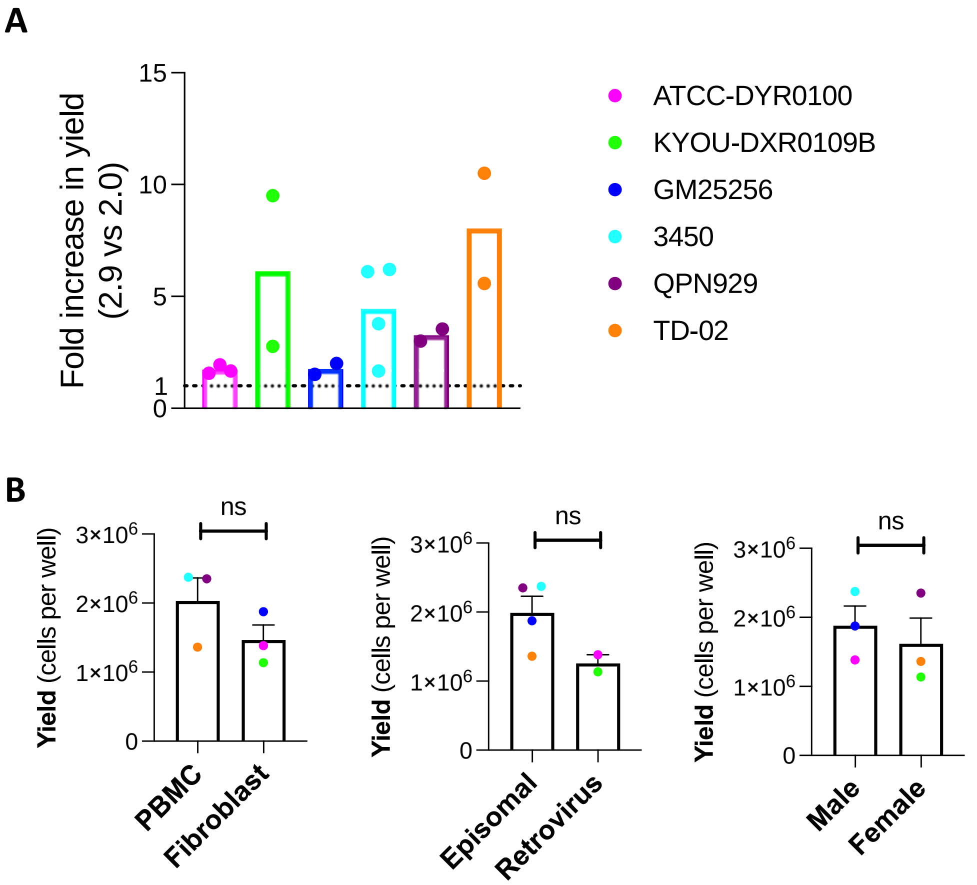

Figure S2. Cell yield of protocols 2.0 and 2.9. iMGL 2.0 and 2.9 were generated side-by-side from six iPSC lines. (A-B) Viable cell yield assessed by Trypan blue exclusion assay. 2-4 batches of differentiation were made per iPSC line. Average of those batches are presented in (B). T-tests were performed in (B), ns = non-significant.

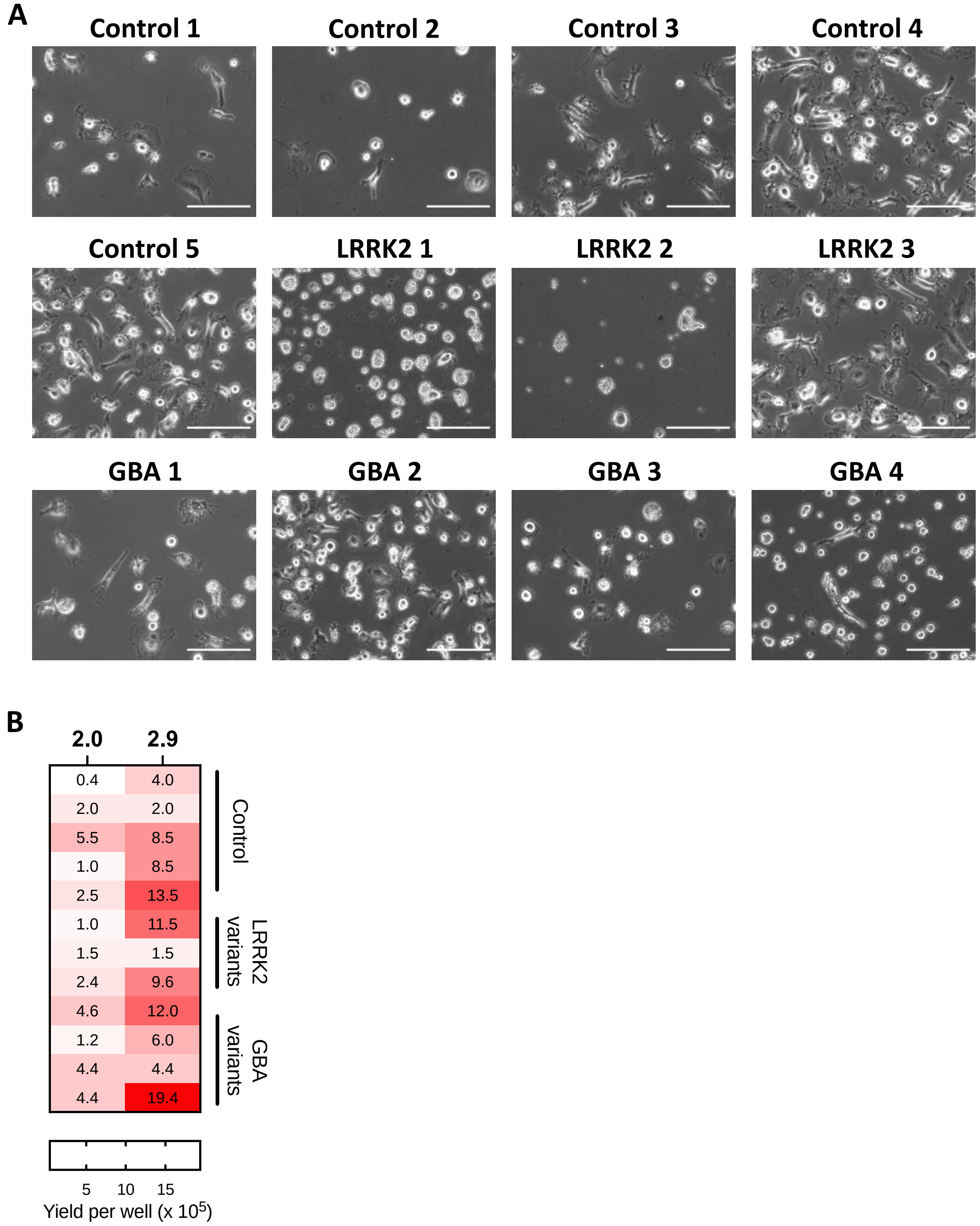

Figure S3. Differentiation of Parkinson's disease patient-derived iPSCs into iMGL 2.9. (A) Phase contrast images of mature iMGL. Scale bar = 150 μm. (B) Viable cell yield per well of a 6-well plate assessed by Trypan blue exclusion assay. n = 1 per cell line.

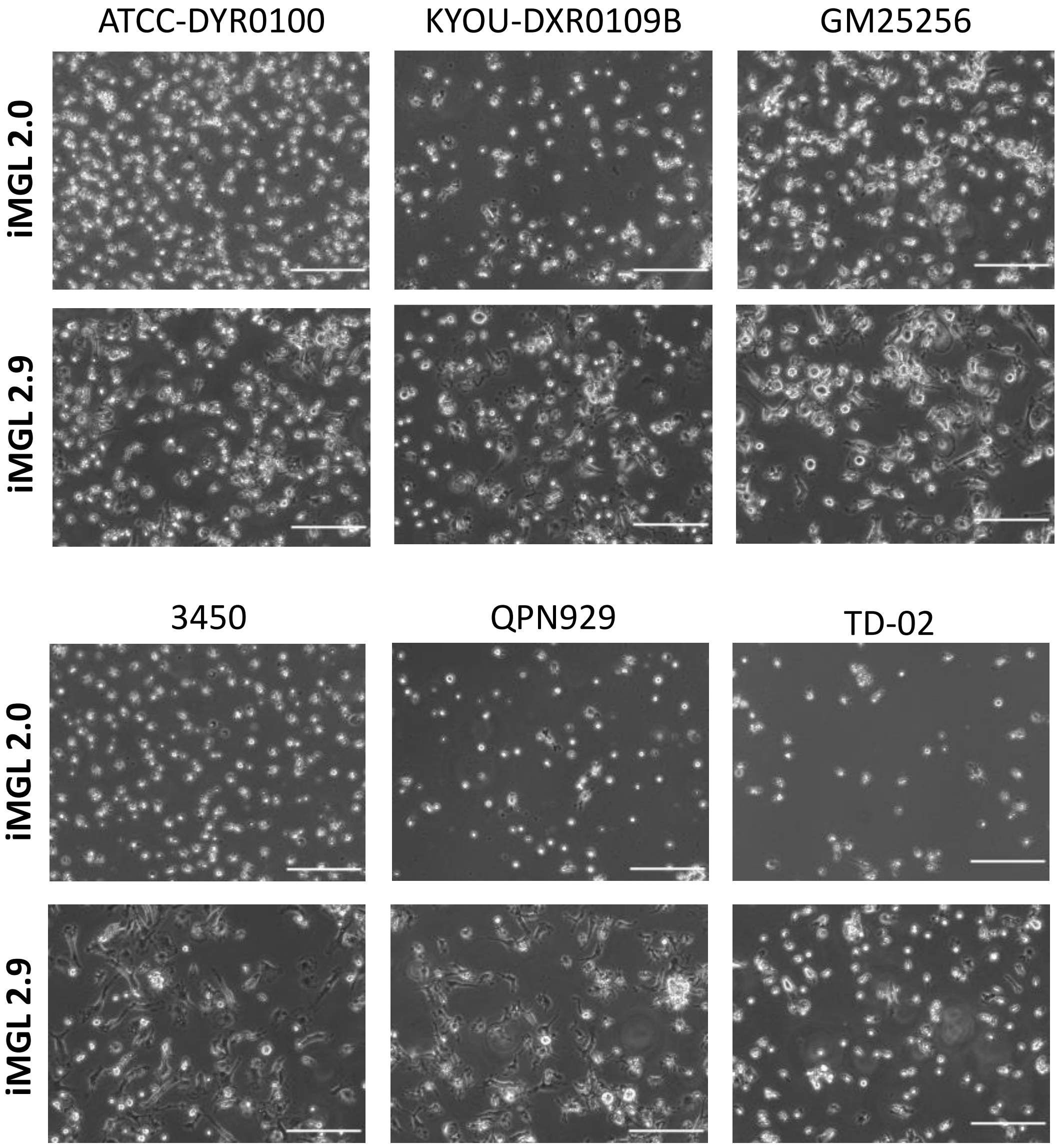

Figure S4. Morphology of iMGL 2.0 and 2.9. iMGL 2.0 and 2.9 were generated side-by-side from six iPSC lines. (A) Viable cell yield assessed by Trypan blue exclusion assay. n = 2-3 per iPSC line. (B) Phase contrast images. Scale bar = 150 μm.

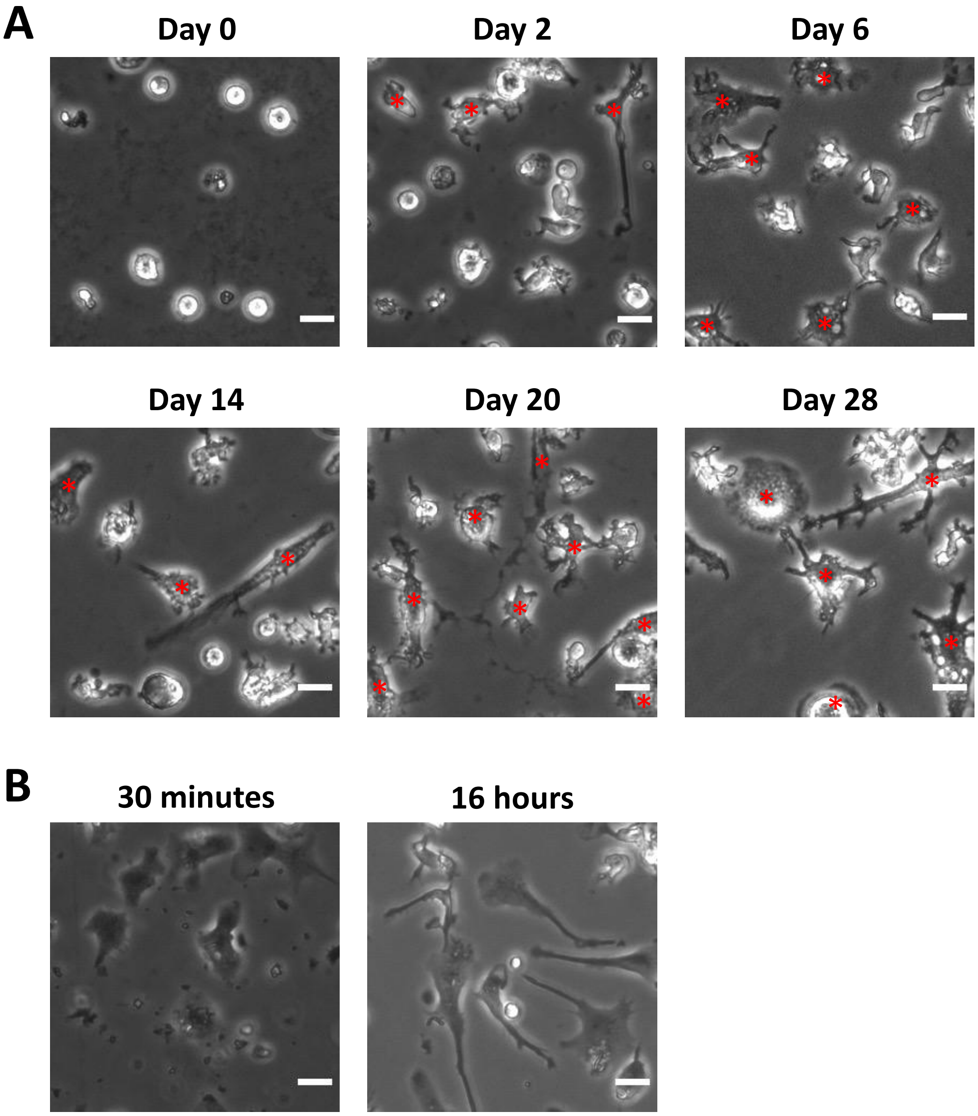

Figure S5. Adhesive property of iMGL 2.9. (A) Phase contrast images of iMGL 2.9 throughout microglial differentiation. Adherent cells are marked by red asterisks. Scale bar = 20 μm. (B) Phase contrast images of mature iMGL 2.9 following replating. Scale bar = 20 μm.

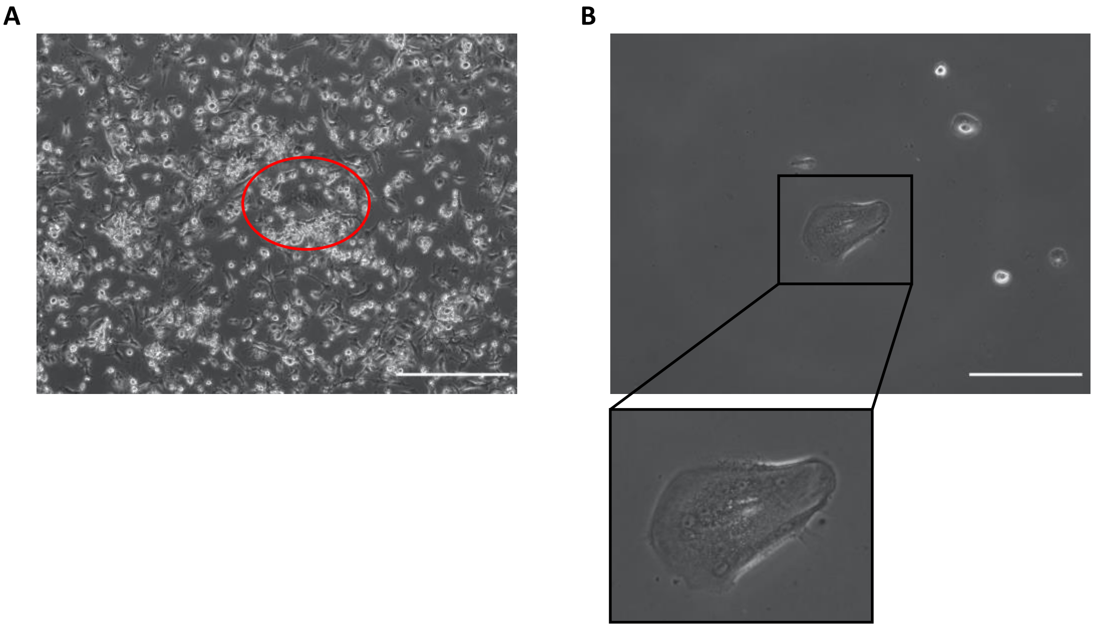

Figure S6. Multinucleated giant cell contaminants. Phase contrast pictures of iMGL 2.9 culture before (A) and after (B) EDTA-mediated cell harvest. Red circle in (A) depicts a multinucleated giant cell underneath mononuclear iMGL. Black square in (B) shows a zoomed-in image of a multinucleated giant cell. Scale bar = 300 μm.

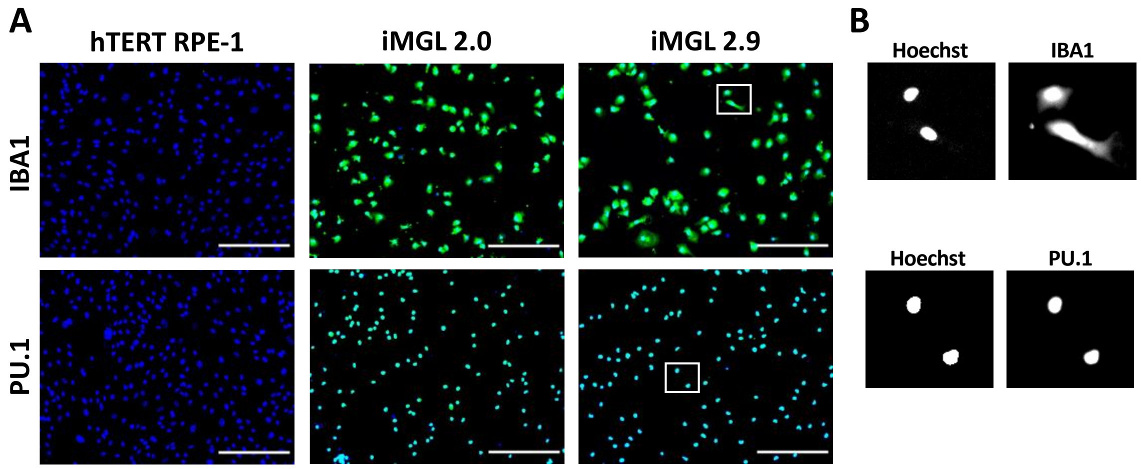

Figure S7. IBA1 and PU.1 immunostaining. iMGL 2.0 and 2.9 were generated side-by-side from the same iPSC line. (A) Representative images. Blue = Hoechst 33342, green = IBA1 or PU.1, scale bar = 200 μm. (B) Zoomed, unmerged images of inserts in (A).

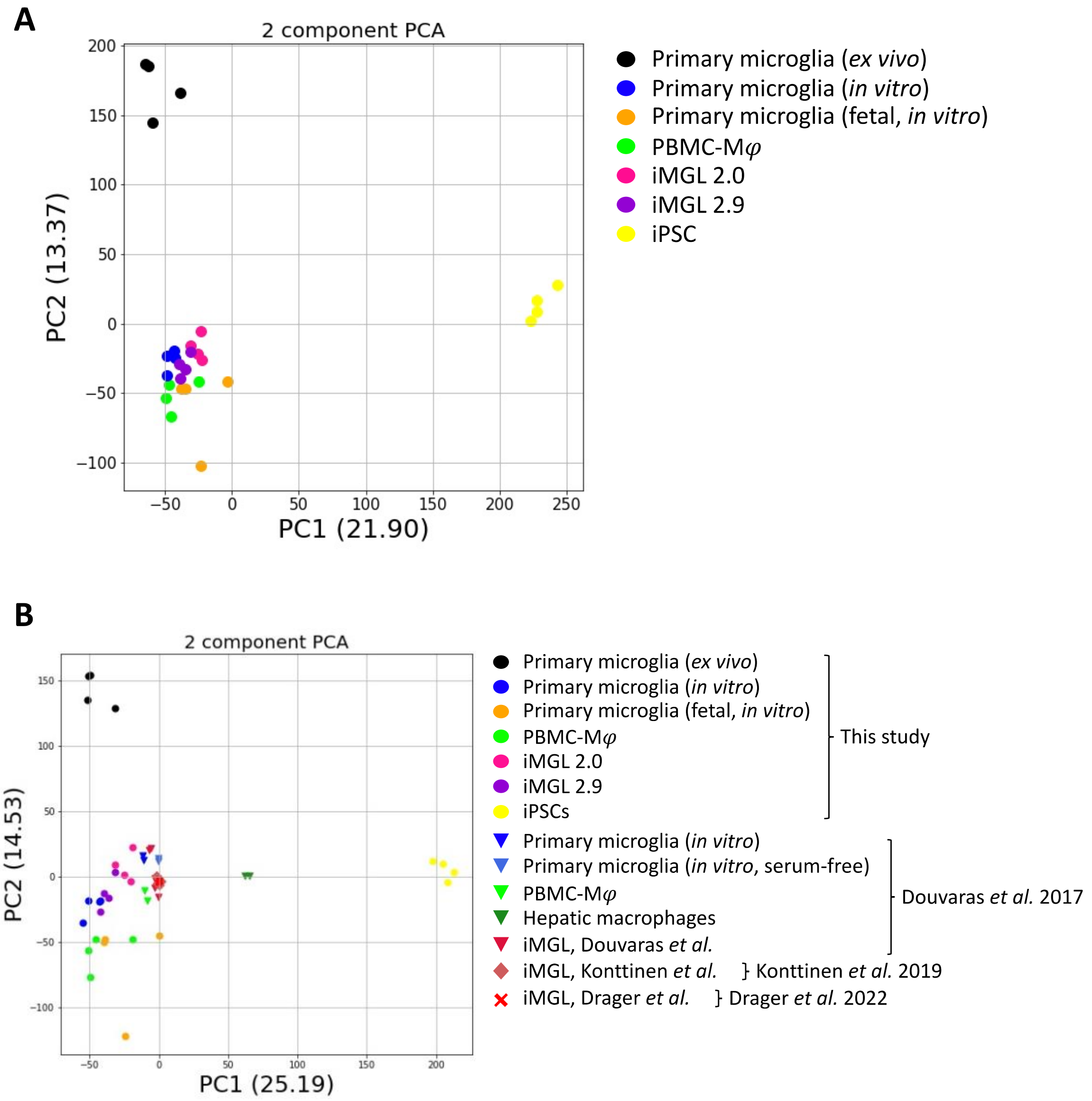

Figure S8. Transcriptome of iMGL 2.0 and 2.9 compared to primary microglia and iPSCs. (A-B) PCA plots of RNA-sequencing data are presented. iMGL 2.0 and 2.9 were differentiated side-by-side from the same four iPSC lines.

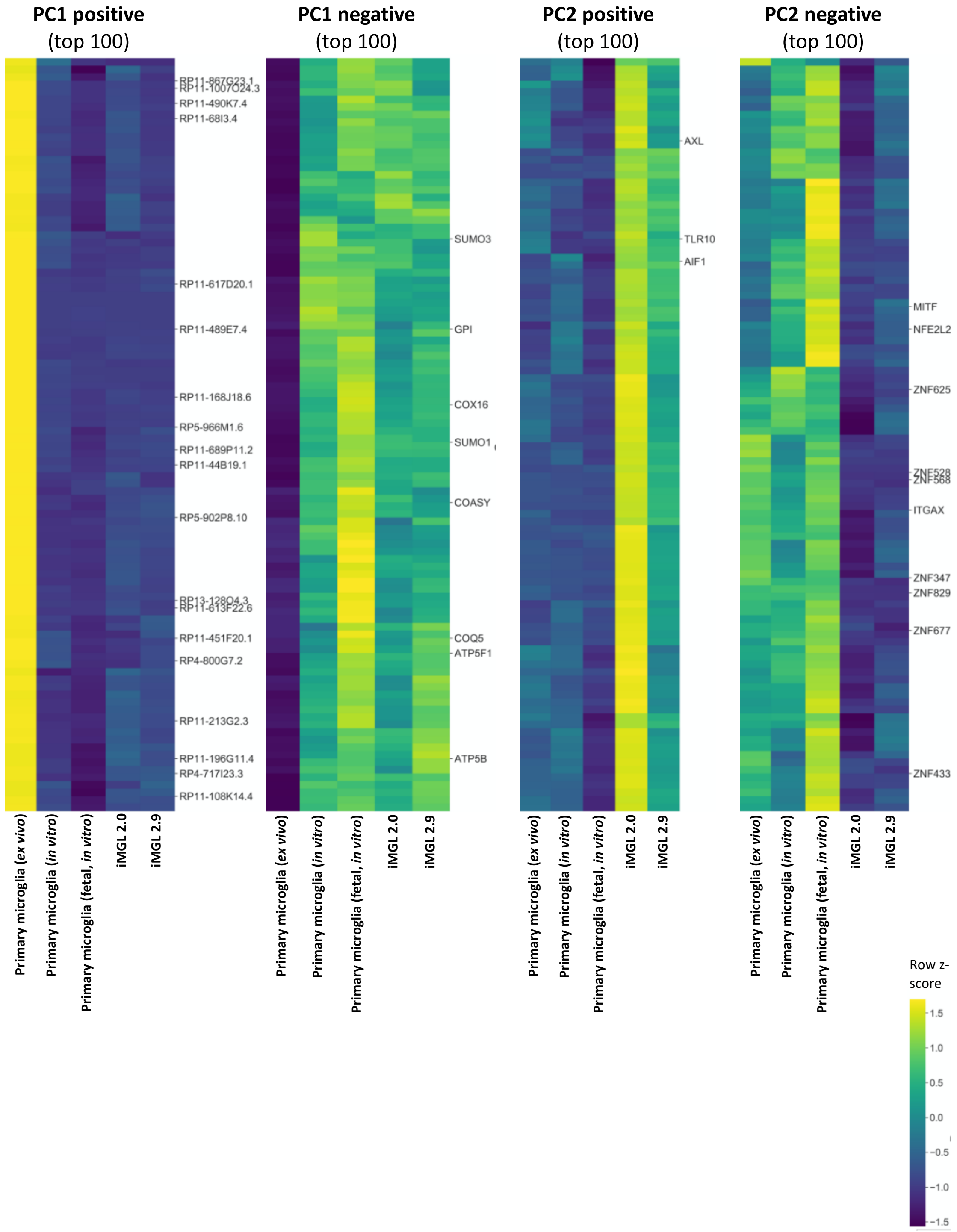

Figure S9. Top genes driving PC1 and PC2 variances. iMGL 2.0 and 2.9 were generated side-by-side from the same iPSC lines. n = 4 donors for primary cells, 4 lines for iMGL**.**

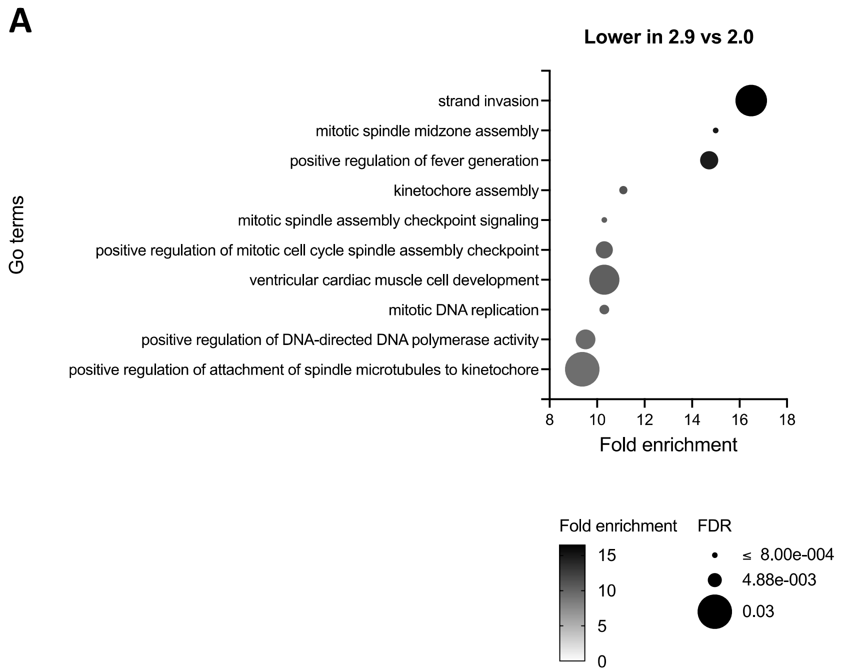

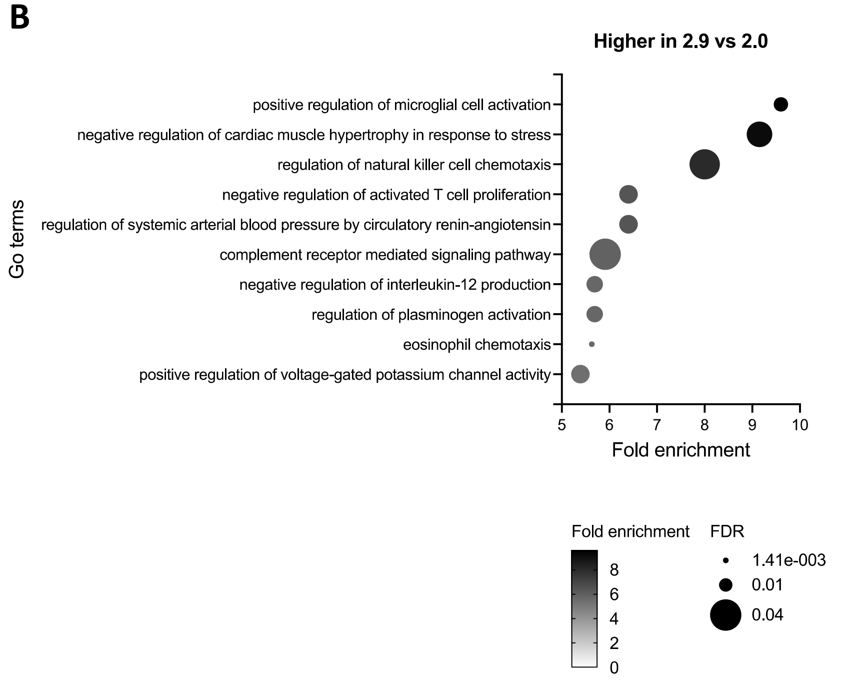

Figure S10. GO term analysis of DEGs identified between iMGL 2.9 and 2.0. iMGL 2.0 and 2.9 were generated side-by-side from the same four iPSC lines. FDR = false discovery rate.

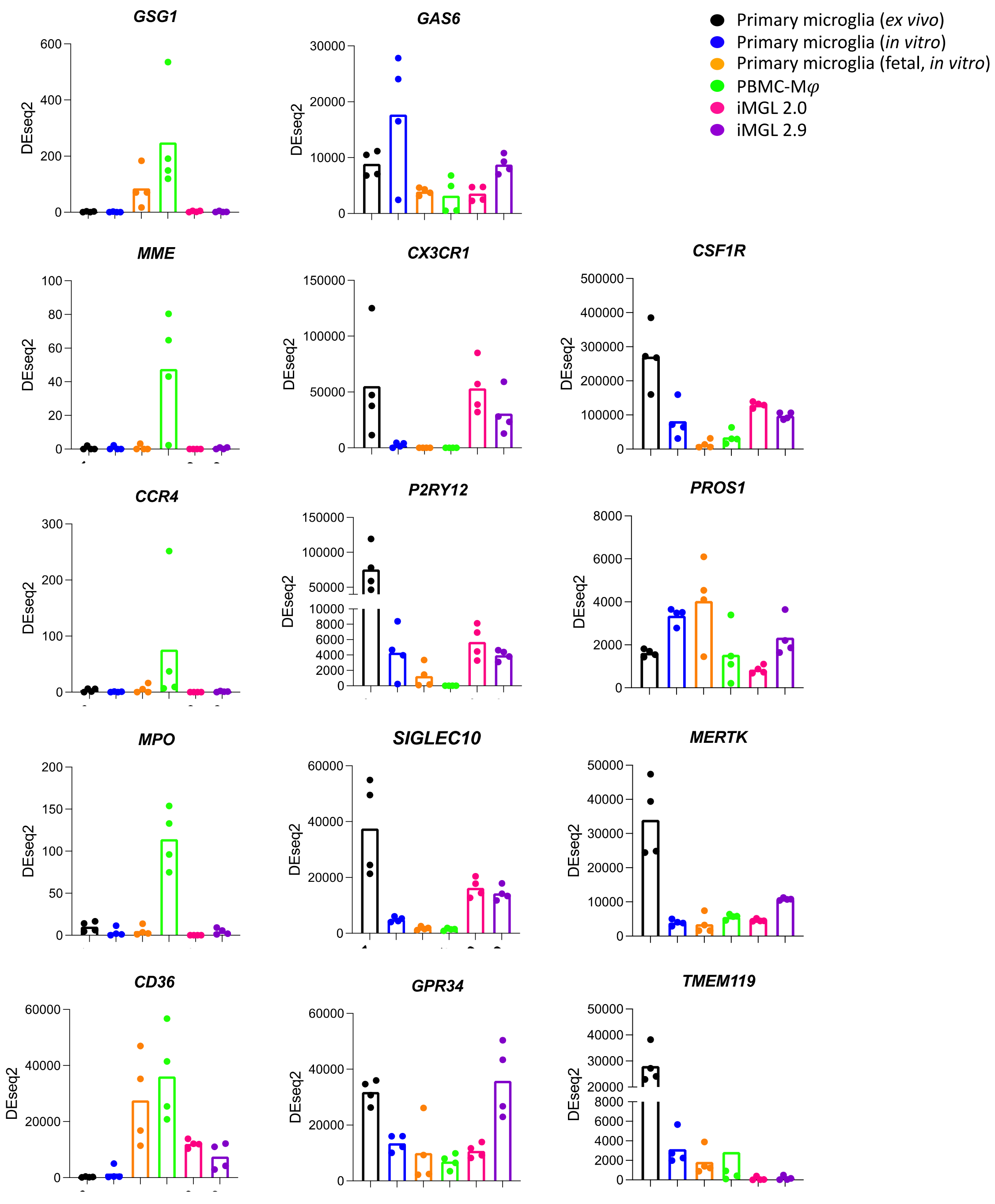

Figure S11. RNAseq analysis of iMGL 2.9 transcriptome. iMGL 2.0 and 2.9 were generated side-by-side from the same iPSC lines. Expression of select macrophage and microglia markers are presented. n = 4 donors for primary cells, 4 lines for iMGL.

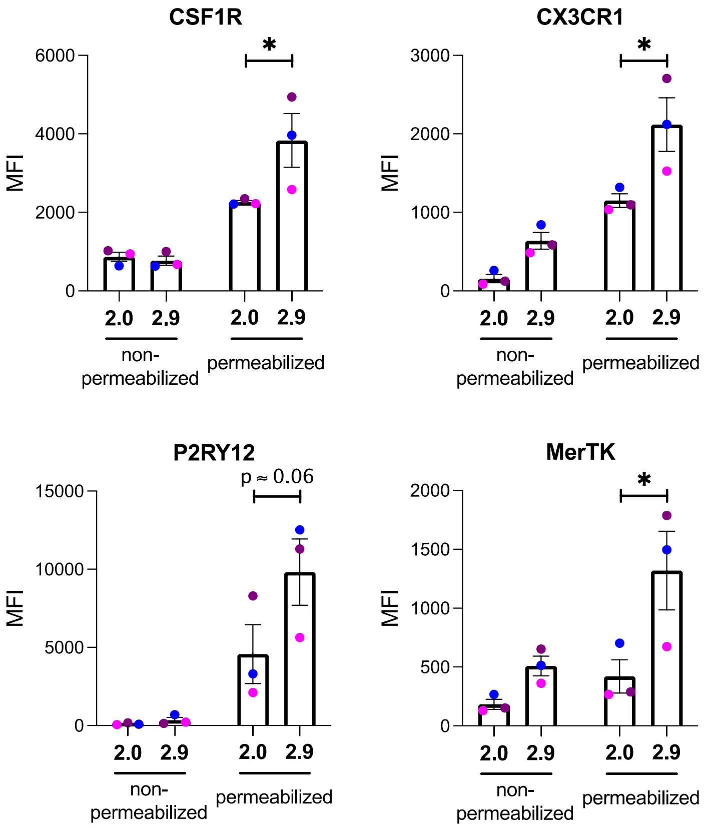

Figure S12. Flow cytometry assessment of microglia markers in iMGL 2.0 and 2.9. iMGL 2.0 and 2.9 were generated side-by-side from the same iPSC lines. Two-way ANOVA were performed, followed by Sidak's post hoc test. n = 3 lines, * $p < 0.05$. MFI = median fluorescence intensity.

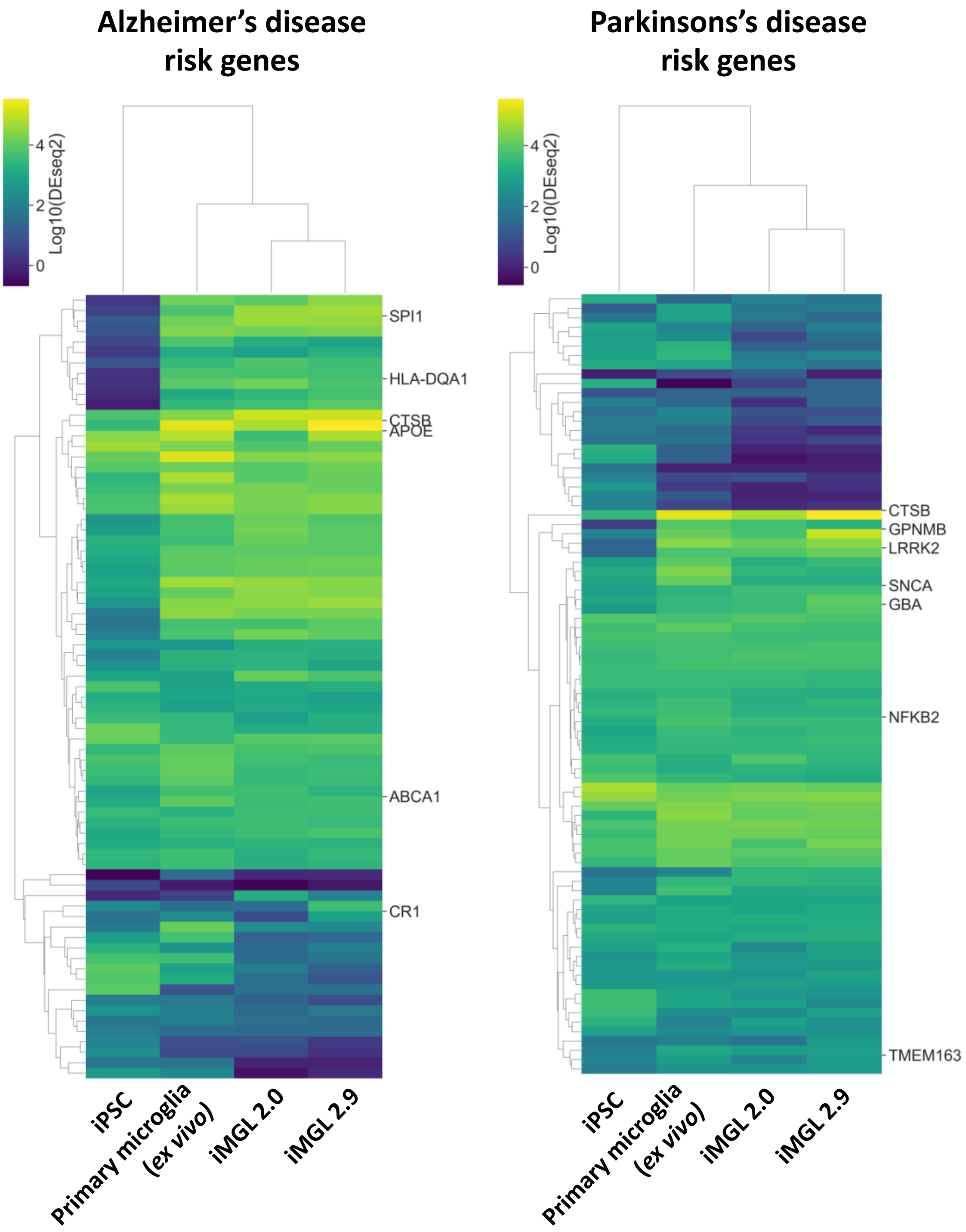

Figure S13. Expression of disease-associated risk genes in iMGL 2.0 and 2.9. iMGL 2.0 and 2.9 were generated side-by-side from the same iPSC lines. n = 4 donors for primary microglia, 4 lines for iMGL and iPSCs.

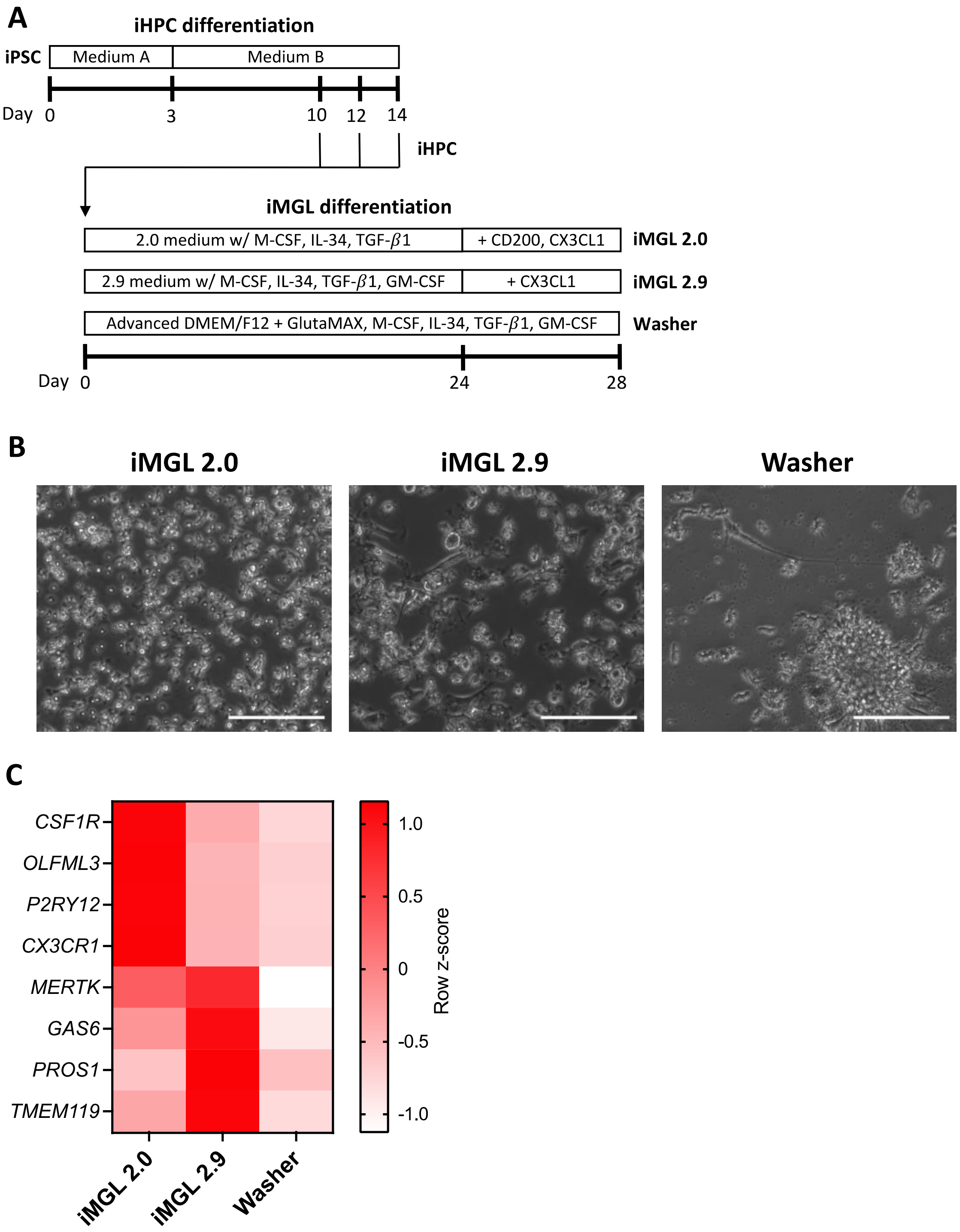

Figure S14. iMGL derived using Washer *et al.*'s microglial differentiation medium. (A) Schematic of the experimental design. iHPCs were subjected to microglial differentiation using the 2.0, the 2.9 or Washer *et al.*'s differentiation media, side-by-side

from the same iPSC lines. Scale bar = 150 μm. (B) Phase contrast images taken at day 28. (C) qRT-PCR assessment of microglia marker genes. n = 2 lines.

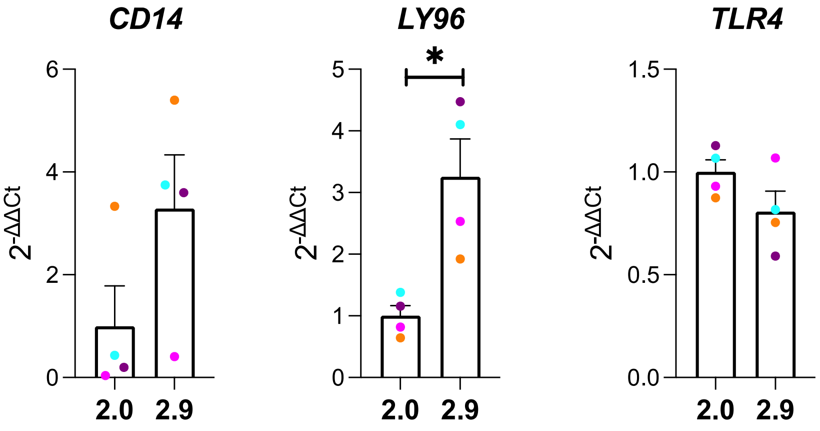

Figure S15. qRT-PCR assessment of *CD14, LY96* and *TLR4* expression in iMGL 2.0 and 2.9. iMGL 2.0 and 2.9 were differentiated side-by-side from the same iPSC lines. T-tests were performed. n = 4 lines, * $p < 0.05$.

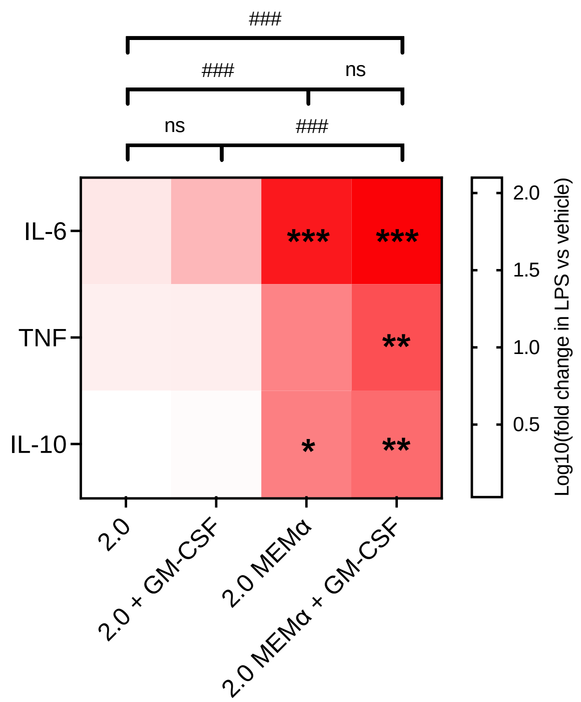

Figure S16. Media-dependent inflammatory response of iMGL to LPS. iMGL were differentiated using the indicated media formulation and then treated with vehicle or LPS (100 ng/mL) for 24 hours. Cytokine secretion in cell supernatants was assessed. A two-way ANOVA was performed, followed by Dunnett's post hoc test (asterisks) and Tukey's post hoc test (hash signs). n = 3 lines, * $p < 0.05$, ** $p < 0.01$, *** $p < 0.001$ vs iMGL 2.0, ns = non-significant, ### $p < 0.001$.

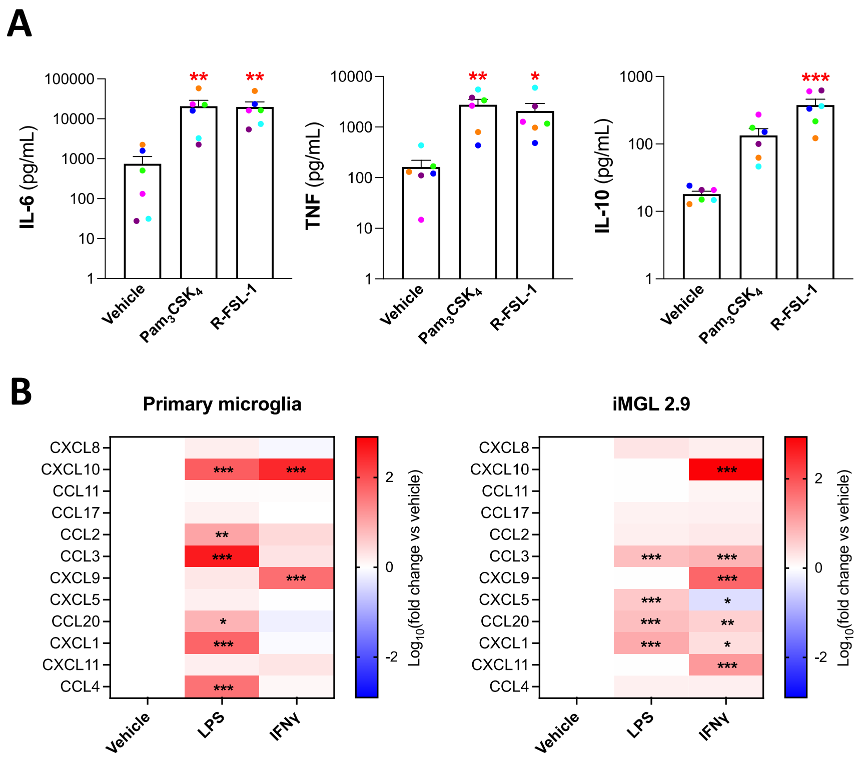

Figure S17. Inflammatory response of iMGL 2.9 to a variety of stimuli. (A) IL-6, TNF and IL-10 concentrations in supernatants from iMGL 2.9 treated with vehicle, Pam_3CSK_4 (100 ng/mL) or R-FSL-1 (250 ng/mL) for 24 hours. One-way ANOVA were performed, followed by Dunnett's post hoc tests. n = 6 lines, * $p < 0.05$, ** $p < 0.01$ vs vehicle. (B) Heatmap representing chemokine secretion from primary microglia or iMGL 2.9 following a 24-hour treatment with LPS (100 ng/mL) or IFNγ (10 ng/mL). Two-way ANOVA were performed, followed by Dunnett's post hoc tests. n = 2 donors for primary microglia and 3 lines for iMGL. * $p < 0.05$, ** $p < 0.01$, *** $p < 0.001$ vs vehicle.
