# Supplementary material for: An adapted protocol to derive microglia from stem cells and its application in the study of CSF1R-related disorders
Source: Mol Neurodegener. 2024 Apr 5;19:31. doi: 10.1186/s13024-024-00723-x (PMC10996091; doi:10.1186/s13024-024-00723-x)
Supplement: Supplementary file 2 — Supplementary Material 2. [file 13024_2024_723_MOESM2_ESM.pdf]

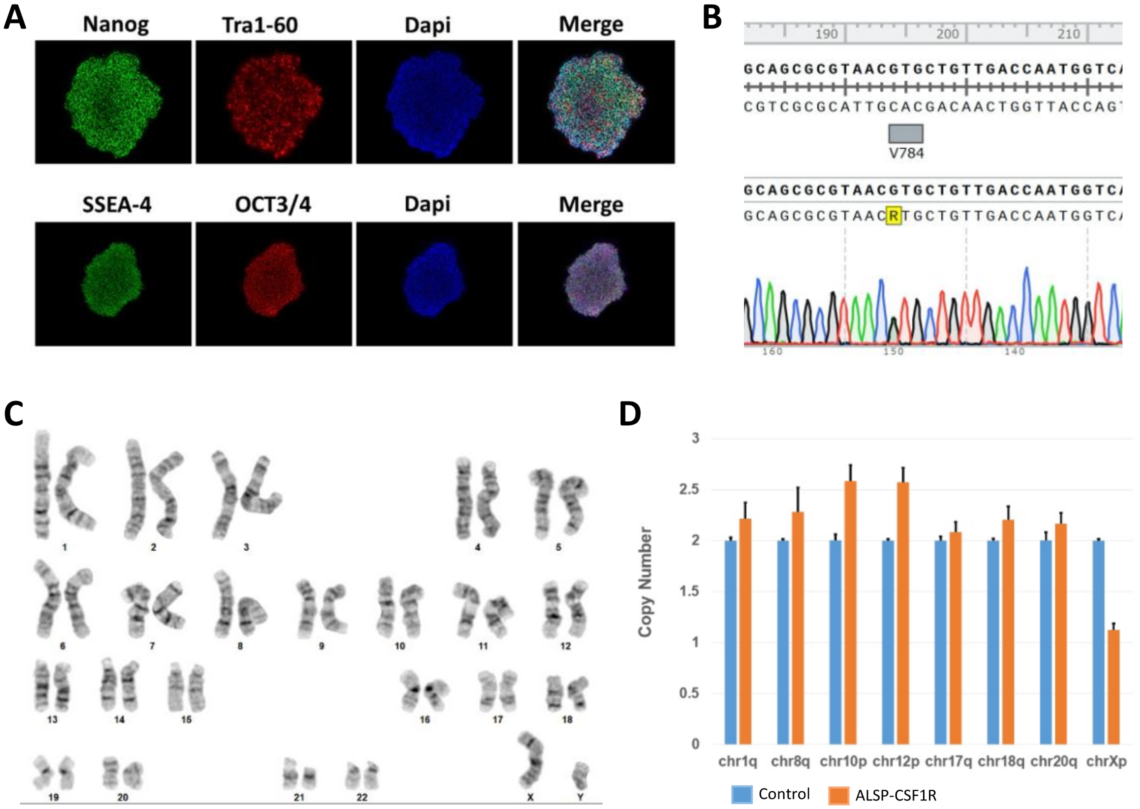

Figure S18. Quality control of the ALSP-CSF1R iPSC line. (A) Immunostaining of pluripotency markers. (B) Chromatogram of the heterozygous c.2350G>A variant in the *CSF1R* gene (shown here at position 150). (C) G-band assay showing normal karyotype of ALSP-CSF1R iPSCs. (D) qPCR-based assessment of copy numbers of chromosomal regions prone to karyotypic abnormalities.

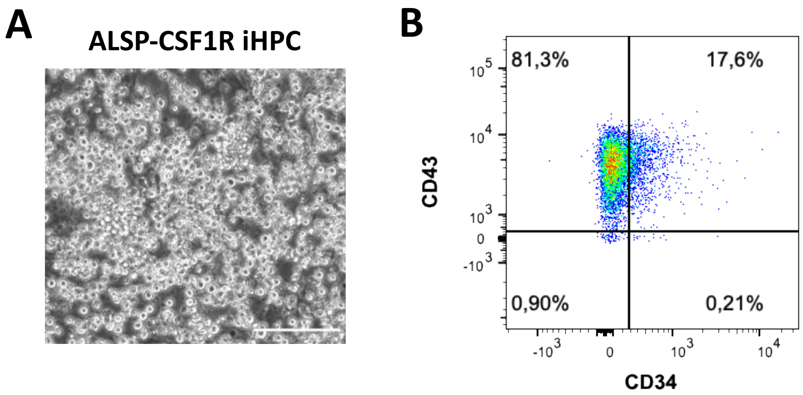

Figure S19. iHPCs generated from ALSP-CSF1R iPSCs. (A) Phase contrast image of iHPC culture on day 12 of differentiation (scale bar = 150 μm). (B) Flow cytometry assessment of CD43 and CD34 in iHPCs on day 12 of differentiation.

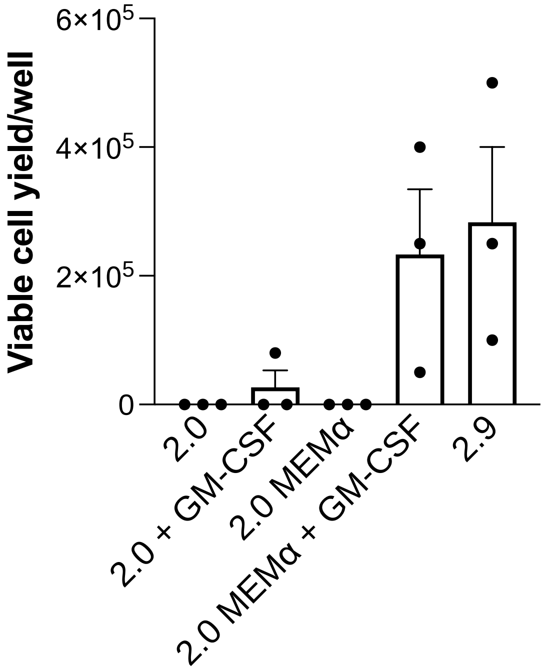

Figure S20. Cell yield following microglial differentiation of ALSP-CSF1R iHPCs using various media formulations. Viable cell yield per well of a 6-well plate assessed by trypan blue exclusion assay. n = 3 differentiation batches.

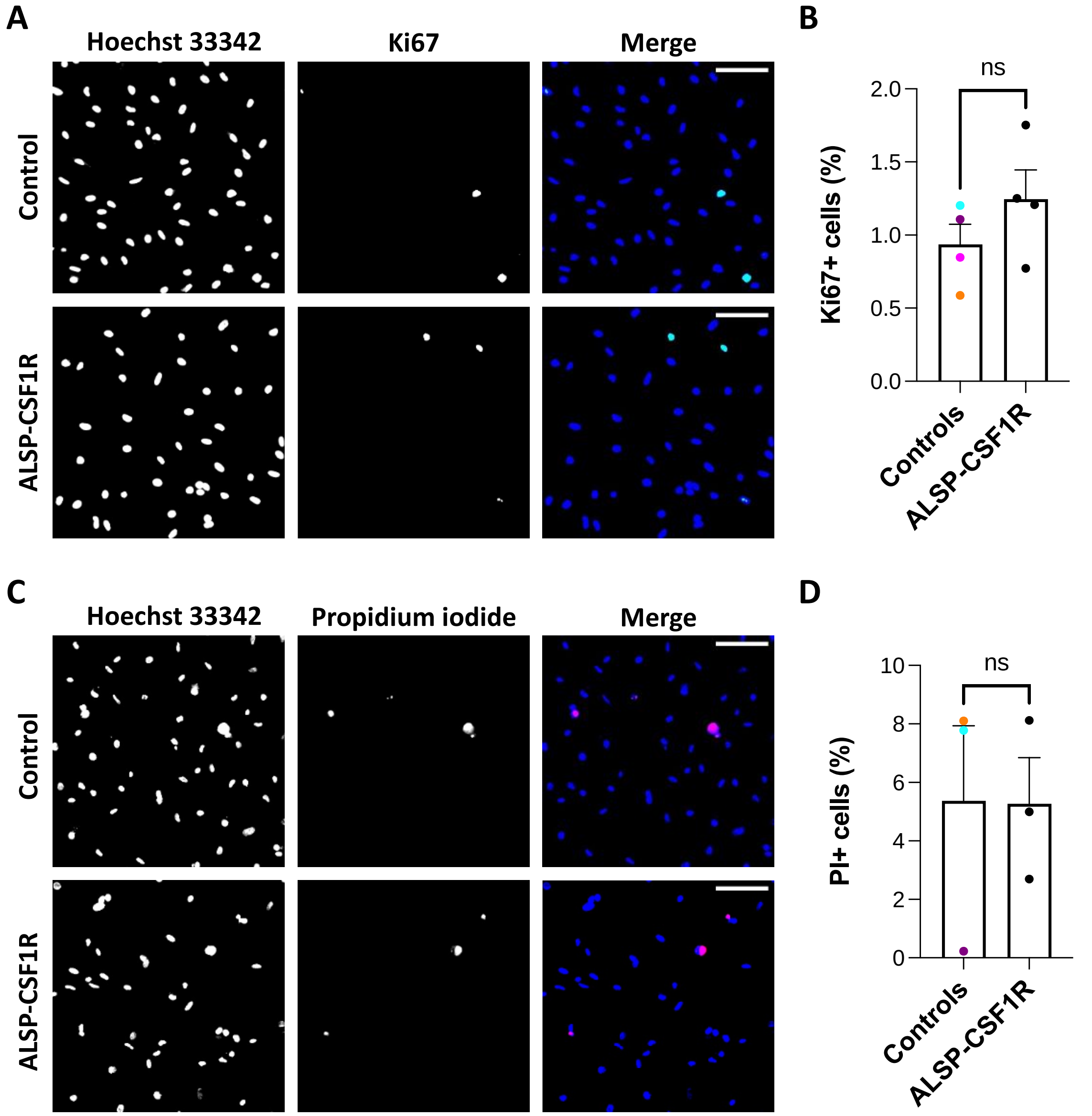

Figure S21. Assessment of proliferation and death rate in mature ALSP-CSF1R iMGL culture. (A) Representative images of Ki67 immunostaining. Scale bar = 50 µm. (B) Quantification of Ki67-immunopositivity. A t-test was performed. n = 3 healthy control lines and 3 batches of a single ALSP patient line, differentiated side-by-side, ns = non-significant. (C) Representative images of propidium iodide staining. Scale bar = 50 µm. (D) Quantification of propidium iodide (PI) -positivity. A t-test was performed. n = 3 healthy control lines and 3 batches of a single ALSP patient line, differentiated side-by-side, ns = non-significant.

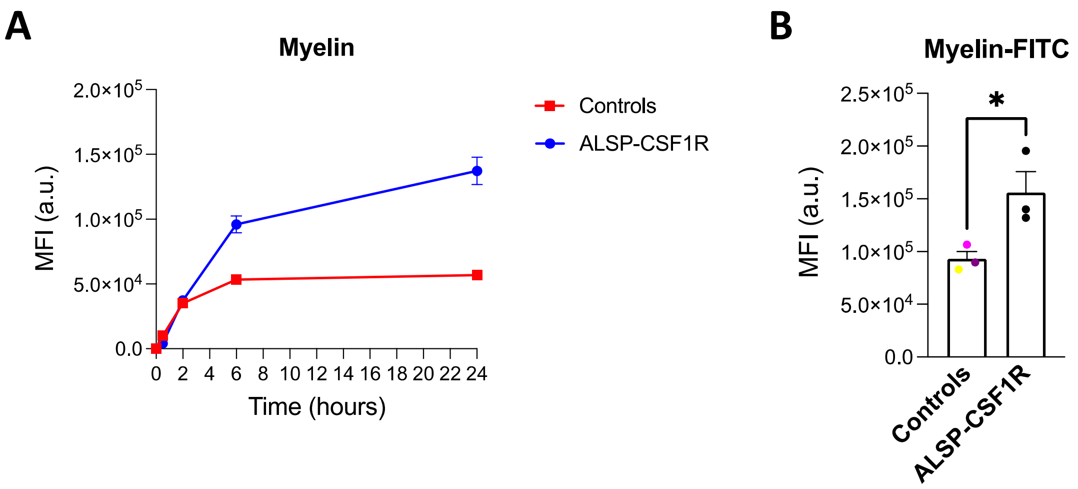

Figure S22. Myelin uptake by ALSP-CSF1R iMGL. iMGL 2.9 were exposed to pHrodo Green^{TM} (A) or FITC (B) -labelled myelin. Cells were counterstained with Hoechst 33342 and mean green fluorescence intensity (MFI) was measured (a.u. = arbitrary unit). (A) Mean +/- standard deviation of three technical replicates are presented. n = 1 healthy control line and 1 batch of an ALSP patient line, differentiated side-by-side. (B) Cells were exposed for three hours. A t-test was performed. n = 3 healthy control lines and 3 batches of a single ALSP patient line, differentiated side-by-side, * $p < 0.05$.

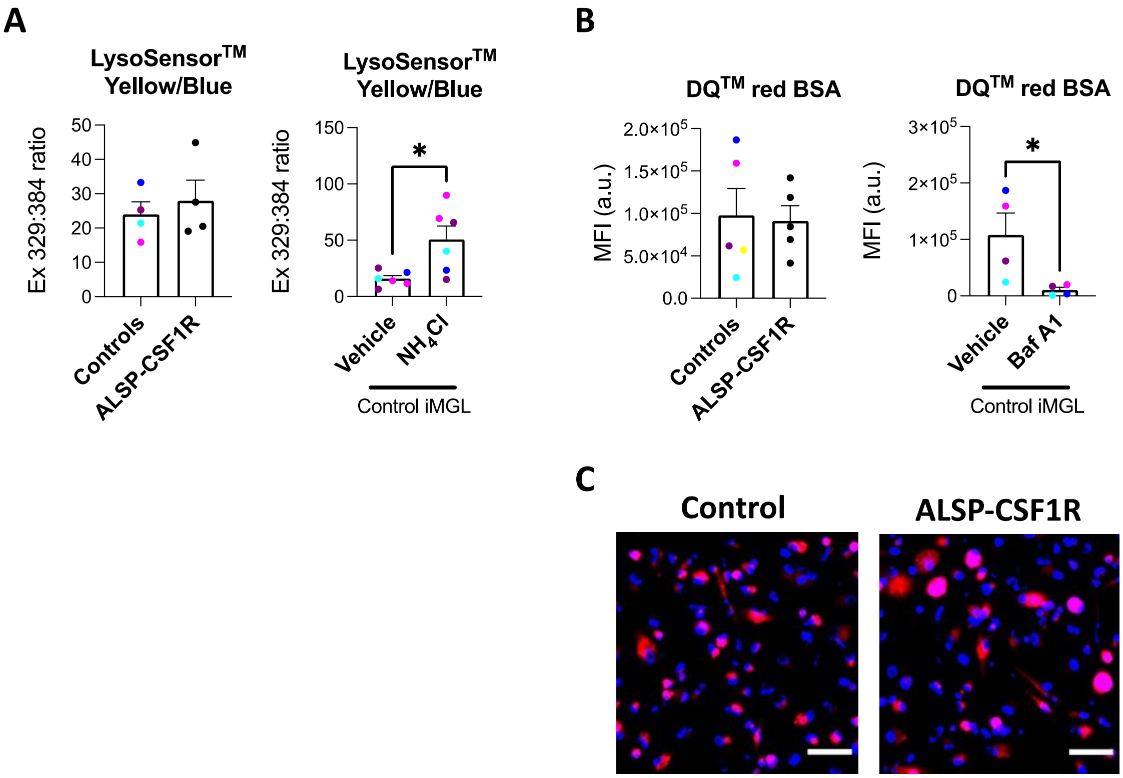

Figure S23. Lysosomal function of ALSP-CSF1R iMGL. All iMGL were generated using the 2.9 protocol. (A) Assessment of lysosomal pH using LysoSensor^{TM} Yellow/Blue. Cell treatment with 50 mM ammonium chloride (NH_4Cl) was used as a positive control. Left panel: n = 4 healthy control lines and 4 batches of a single ALSP patient line, differentiated side-by-side. Right panel: n = 6 batches from 4 healthy control lines. T-tests were performed, * $p < 0.05$. (B-C) Assessment of lysosomal protease activity using DQ^{TM} red BSA. (B) Cell treatment with bafilomycin A1 (Baf A1, 100 nM) was used as a control. MFI = mean fluorescence intensity, a.u. = arbitrary unit. Left panel: n = 5 healthy control lines and 5 batches of a single ALSP patient line, differentiated side-by-side. Right panel: T-tests were performed. n = 4 healthy control lines, * $p < 0.05$. (C) Representative fluorescence images. Scale bar = 50 μm.

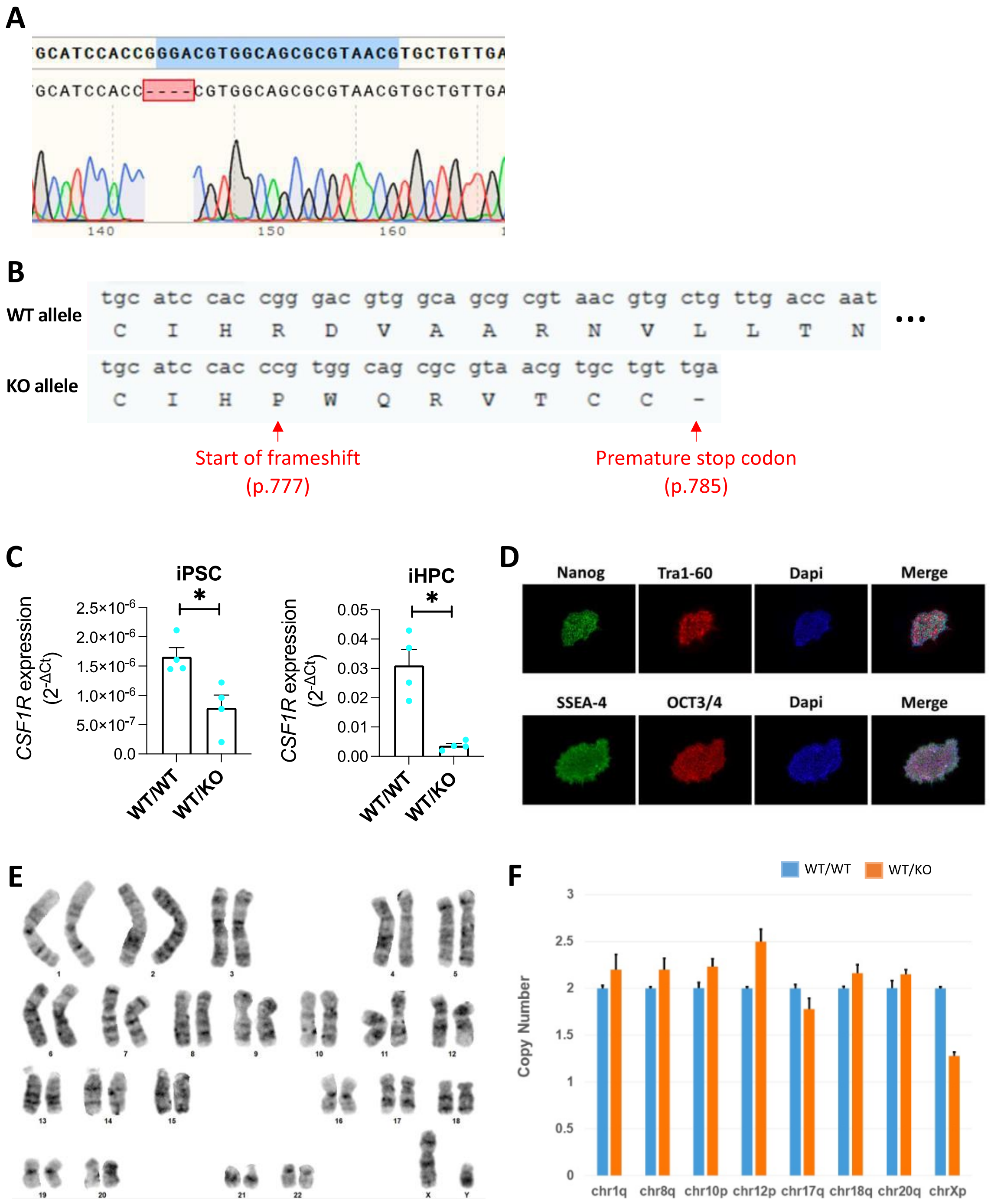

Figure S24. Quality control of the *CSF1R*$^{WT/KO}$ iPSC line. (A) Chromatogram of the heterozygous 4-base pair deletion (c.2330_2333del) in *CSF1R*. Shaded area of the sequence represents the position recognized by the guide RNA. (B) Protein sequence predicted using Expasy Translate tool (https://web.expasy.org/translate/). (C) qRT-PCR

assessment of *CSF1R* expression in iPSCs and iHPCs. Mann-Whitney tests were performed. n = 4 differentiation batches, * $p < 0.05$. *CSF1R*$^{WT/WT}$ and *CSF1R*$^{WT/KO}$ cells were differentiated side-by-side into iHPCs. (D) Immunostaining of pluripotency markers in iPSCs. (E) G-band assay showing normal karyotype of ALSP-CSF1R iPSCs. (F) qPCR-based assessment of copy numbers of chromosomal regions prone to karyotypic abnormalities in iPSCs.

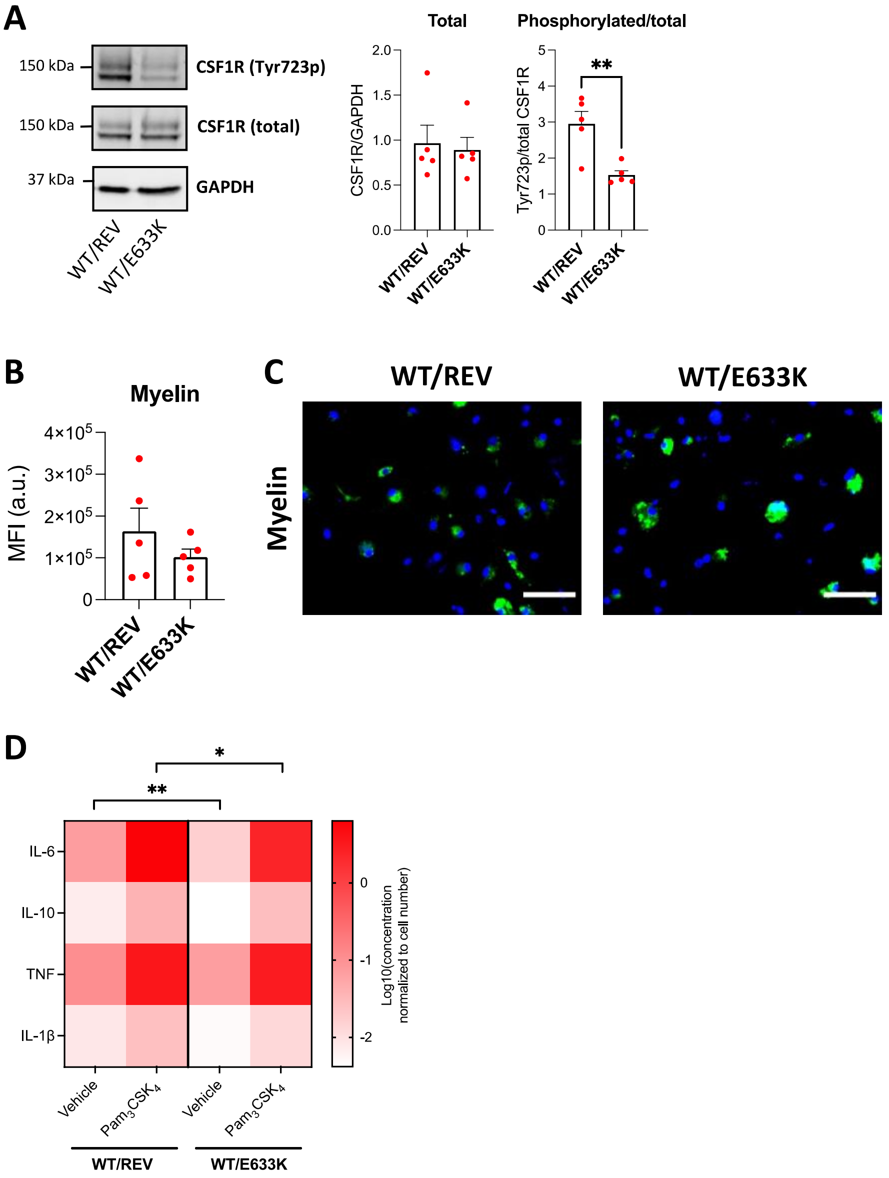

Figure S25. Functional features of *CSF1R*$^{WT/E633K}$ iMGL compared to their isogenic controls. *CSF1R*$^{WT/REV}$ and *CSF1R*$^{WT/E633K}$ iPSCs were differentiated into iMGL side-by-side using the 2.9 protocol. (A) Western blot assessment of CSF1R and its tyrosine 723-phosphorylated form, and GAPDH. A t-test was performed. n = 5 differentiation batches, ** $p < 0.01$. (B-C) iMGL were exposed for three hours to pHrodo™ Green-labelled myelin debris. Cells were counterstained with Hoechst 33342 and mean green fluorescence intensity (MFI) was measured (a.u. = arbitrary unit). (B) Quantification of MFI. A t-test was performed. n = 5 differentiation batches. (C) Representative fluorescence images. Scale bar = 50 μm. (D) Cytokine concentrations measured in cell supernatants following a

24-hour treatment with vehicle or Pam_3CSK_4 (100 ng/mL). Two-way ANOVA were performed, followed by Tukey's post hoc test. n = 5 differentiation batches, * $p < 0.05$, ** $p < 0.01$.

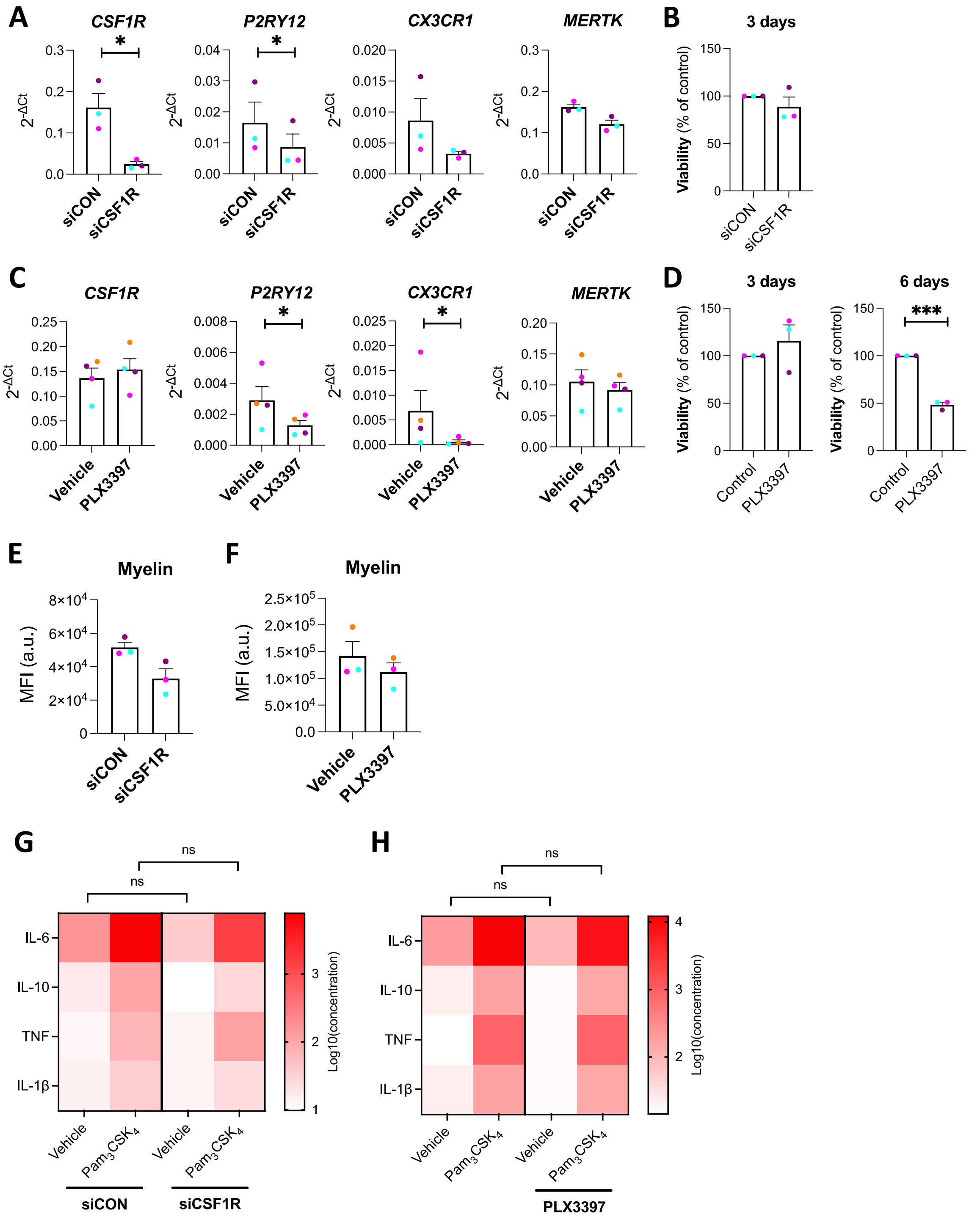

Figure S26. Effect of CSF1R knockdown or inhibition on mature iMGL. Healthy control, mature iMGL were generated following the 2.9 protocol. (A) Microglia marker expression assessed by qRT-PCR in iMGL three days after siCON or siCSF1R transfection.

T-tests were performed. n = 3 lines, * $p < 0.05$. (B) Viability of iMGL three days after siCON or siCSF1R transfection. A Mann-Whitney test was performed. n = 3 lines. (C) Microglia marker expression assessed by qRT-PCR in iMGL following a 3-day treatment with PLX3397 (1 μM, every other day). T-tests was performed. n = 4 lines, * $p < 0.05$. (D) Viability of iMGL following 3-day and 6-day treatments with PLX3397 (1 μM, every other day). Mann-Whitney tests were performed. n = 3 lines, *** $p < 0.001$. (E-F) iMGL were exposed for three hours to pHrodo™ Green-labelled myelin debris. Cells were counterstained with Hoechst 33342 and mean green fluorescence intensity (MFI) was measured (a.u. = arbitrary unit). (E) Myelin uptake by iMGL three day after siCON or siCSF1R transfection. (F) Myelin uptake by iMGL following a 3-day treatment with PLX3397 (1 μM, every other day). A t-test was performed. n = 3 lines. (G-H) Cytokine concentrations measured in cell supernatants following a 24-hour treatment with Pam_3CSK_4 (100 ng/mL). Two-way ANOVA were performed, followed by Tukey's post hoc test. ns = non-significant. (G) Cells were used three days after transfection with siCON or siCSF1R; n = 3 lines. (H) Cells were treated for three days with PLX3397 (1 μM, every other day); n = 4 lines.

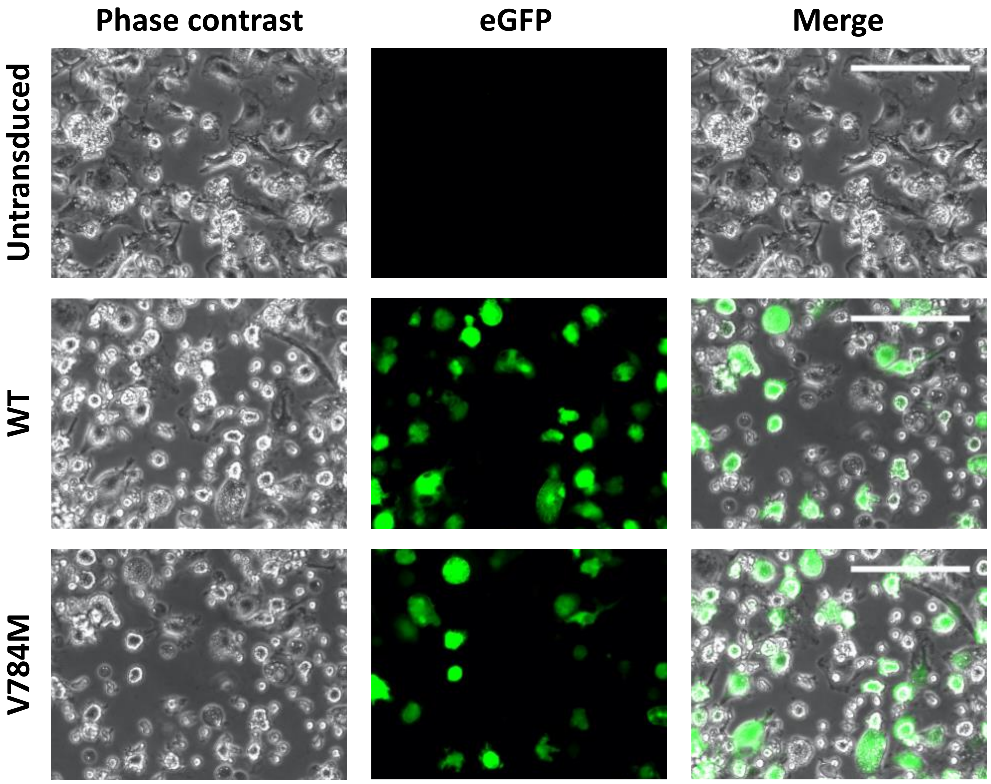

Figure S27. Phase contrast and green fluorescence images of transduced ALSP-CSF1R iMGL. eGFP and either WT or V784M CSF1R were stably co-expressed in ALSP-CSF1R iHPCs using lentiviruses and cells were differentiated into iMGL following the 2.9 protocol. Images were taken on day 28 of microglial differentiation. Scale bar = 150 μm.

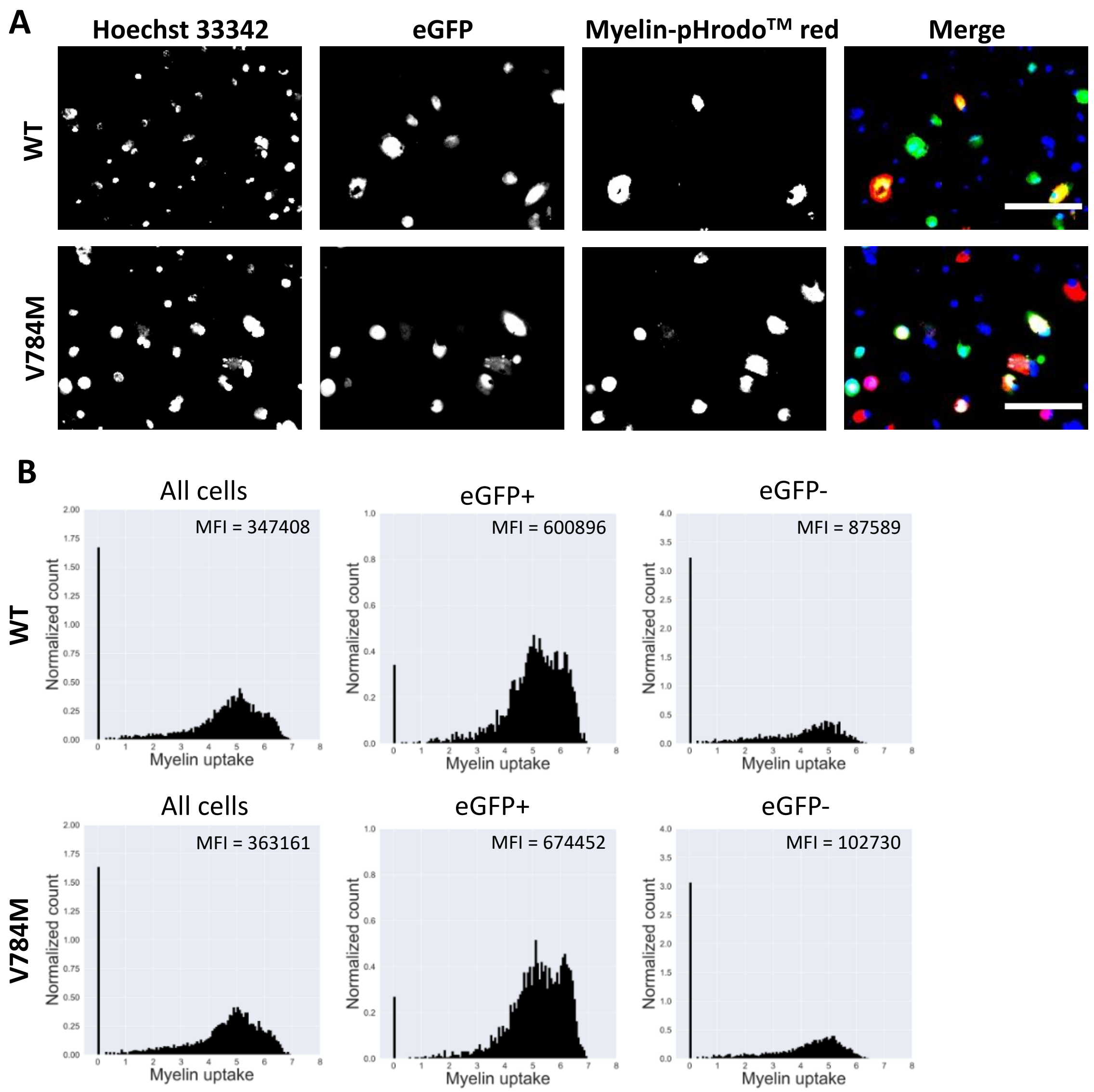

Figure S28. Effect of lentivirus-induced stable WT and V784M CSF1R expression on myelin uptake by ALSP-CSF1R iMGL. eGFP and either WT or V784M CSF1R were stably co-expressed in ALSP-CSF1R iHPCs using lentiviruses and cells were differentiated into iMGL following the 2.9 protocol. iMGL were exposed to myelin pHrodo^{TM} Red for 3 hours and counterstained with Hoechst 33342. (A) Representative fluorescence images. Scale bar = 100 µm. (B) Measurement of red fluorescence intensity. MFI = mean fluorescence intensity. n = 1 differentiation batch.
